# Screening of a Novel *Lactiplantibacillus plantarum* MMB-05 and *Lacticaseibacillus casei* Fermented Sandwich Seaweed Scraps: Chemical Composition, In Vitro Antioxidant, and Volatile Compounds Analysis by GC-IMS

**DOI:** 10.3390/foods11182875

**Published:** 2022-09-16

**Authors:** Tengqi Gao, Jinling Chen, Jie Xu, Han Gu, Pengpeng Zhao, Wenbin Wang, Saikun Pan, Yang Tao, Hongli Wang, Jie Yang

**Affiliations:** 1 Jiangsu Key Laboratory of Marine Bioresources and Environment/Jiangsu Key Laboratory of Marine Biotechnology, Jiangsu Ocean University, Lianyungang 222005, China; 2 Co-Innovation Center of Jiangsu Marine Bio-industry Technology, Jiangsu Ocean University, Lianyungang 222005, China; 3 Anqiu Agricultural Product Quality and Safety Management Service Center, AnQiu 262100, China; 4 College of Food Science and Technology, Nanjing Agricultural University, 1 Weigang, Nanjing 210095, China

**Keywords:** lactic acid bacteria, sandwich seaweed scraps, *Porphyra yezoensis* sauce, volatile compounds, GC-IMS

## Abstract

Lactic acid fermentation is a promising method for developing sandwich seaweed scraps. The objectives of this study were to investigate the effect of fermentation with *Lactiplantibacillus plantarum* MMB-05, *Lactiplantibacillus casei* FJAT-7928, mixed bacteria (1:1, *v/v*) and control on the physicochemical indexes, in vitro antioxidant activity, and volatile compounds of *Porphyra yezoensis* sauce. Sensory evaluation was also performed. The results indicated that all lactic acid bacteria strains grew well in *P. yezoensis* sauce after 72 h of fermentation, with the viable cell counts of *L. plantarum* MMB-05 exceeding 10.0 log CFU/mL, the total phenolic content increasing by 16.54%, and the lactic acid content increasing from 0 to 44.38 ± 0.11 mg/mL. Moreover, the metabolism of these strains significantly increased the content of umami, sweet and sour free amino acids in *P. yezoensis* sauce. The total antioxidant capacity of *L. plantarum* MMB-05, *L. casei* FJAT-7928, mix and control groups increased by 594.59%, 386.49%, 410.27%, and 287.62%, respectively. Gas chromatography-ion mobility spectrometry (GC-IMS) analysis suggested that aldehydes and ketones accounted for the largest proportion, and the relative contents of acids and alcohols in *P. yezoensis* sauce increased significantly after lactic acid bacteria fermentation. In addition, the analysis of dynamic principal component analysis (PCA) and fingerprinting showed that the volatile components of the four treatment methods could be significantly distinguished. Overall, the *L. plantarum* MMB-05 could be recommended as an appropriate starter for fermentation of sandwich seaweed scraps, which provides a fundamental knowledge for the utilization of sandwiched seaweed scraps.

## 1. Introduction

With the improvement of people’s living standards, consumers are gradually inclined to choose convenient, nutritious, and functional foods, and pay more attention to the quality of food [1]. Sandwiched seaweed is one of the very popular seafood products with high commercial prospects. It is mainly made of *Porphyra yezoensis*, with crisp and fragrant product characteristics, and is a new product in recent years. Laver is a kind of important cultivated red seaweed with high nutritional value and biotechnology potential. It is rich in protein, carbohydrates, amino acids, and polyphenols, which mainly grown in China, Japan, and South Korea, and had an annual harvest of 0.222 million tons in 2020 [2,3,4]. Among them, *P. yezoensis* is one of the most economical seaweed species in China [5]. However, in the process of making sandwich seaweed from *P. yezoensis*, a lot of scraps will be produced, resulting in unnecessary waste. Therefore, there is an urgent need to make full use of these scraps.

In recent years, lactic acid bacteria fermented food has attracted extensive attention, which could improve the nutrition and sensory quality of food, enrich human life, and improve human well-being. Hadj et al. [6] utilized a method of fermenting waste vanilla beans to recover vanillin and other valuable aromatic compounds through metabolic transformation of lactic acid bacteria (LAB). Lee et al. [7] used waste coffee grounds as raw material for lactic acid fermentation, which improved the conversion rate of sugar. *Lactiplantibacillus plantarum* and *Lacticaseibacillus casei* are common lactic acid bacteria in food fermentation, and are widely used as food starter cultures [8,9]. In particular, *L. plantarum* has the potential to improve food flavor and is the preferred strain for fermented foods as it could use carbohydrates to produce organic acids, and contains enzymes that could degrade peptides into small peptides and free amino acids. It also produces a variety of volatile and non-volatile components, thus giving the product a unique flavor. There have been a number of recent reports and studies examining lactic acid bacteria fermented foods, most of which have focused on meat, fish sauce, grain, fruit, vegetables, and dairy products [10,11,12,13,14,15], while relatively few studies have been conducted on lactic acid bacteria fermented algal products. Gradually, reports of lactic acid bacteria fermentation of *P. yezoensis* sauce began to appear. Uchida et al. [16] studied how *Tetragenococcus halophilus* could grow *P. yezoensis* sauce and hoped it could be used to produce fermented seaweed sauce. Uchida et al. [17] evaluated the characterization of halophilic lactic acid bacteria and found that seaweed paste was rich in total nitrogen compounds and potassium, and had a unique free amino acids composition. To our best knowledge, there has been no previous study on physicochemical properties, in vitro antioxidant activity, and volatile compounds of *P. yezoensis*. Gas chromatography-ion mobility spectrometry (GC-IMS) technology is a recently developed analytical technique. The technology combines the advantages of the high resolution of GC and the high sensitivity of ion mobility spectrometry, and without any special sample preparation it could be used for rapid analysis of trace volatile organic compounds in samples [18,19]. Currently, there is an increasing number of researchers interested in the analysis of volatile components in lactic acid bacteria fermentation products for food analysis [20,21].

In previous research, our research group found that *L. casei* FJAT-7928, *Streptococcus thermophilus* FJAT-46738, and *Limosilactobacillus fermentum* FJAT-46744, could ferment sandwich seaweed scraps to prepare *P. yezoensis* sauce, resulting in high value utilization of resources [22]. However, there is no in-depth understanding of *L. plantarum* fermented sandwich seaweed scraps and the long fermentation time is not conducive to industrial production. Thus, this paper screened a strain of *L. plantarum* and compared it with the previous *L. casei* fermented *P. yezoensis* sauce. The fermentation characteristics, in vitro antioxidant activity, sensory evaluation, and volatile flavor of the two strains were evaluated. These results may provide theoretical guidance for the efficient processing of *P. yezoensis* sauce, thus improving the utilization rate and the value of sandwich seaweed resources.

## 2. Materials and Methods

### 2.1. Isolation, Purification, and Identification of L. Plantarum MMB-05

A 10 mL sample of pickle brine brought from southeastern Guizhou Province was incubated with 90 mL MRS liquid medium (Beijing Luqiao Technology Co., LTD, Beijing, China) based on flasks at 37 °C for 48 h, then centrifuged at 14,130× *g* for 15 min at 4 °C, before discarding the sediment and taking out the supernatant. The supernatant was diluted to an appropriate gradient using 0.9% sterile normal saline, and 100 μL was absorbed into MRS solid medium for coating and incubated at 37 °C for 48 h, and 10% colonies of different forms were randomly removed from each plate. The underlined screening was repeated on plates, individual colonies were selected, with further underlined isolation and purification. The isolated strains were stored in 40% (volume fraction) glycerol at −80 °C for further use.

To screen LAB strains for further study, 20 g of dried seaweed sandwich scraps (Lianyungang Wende Food Co., Ltd. Lianyungang, Jiangsu, China) were weighed and 30 mL of distilled water was added. The seaweed sandwich scraps were sterilized at 121 °C for 30 min prior to fermentation. After cooling to room temperature, 1.5 mL of bacterial liquid was subsequently added to the seaweed sandwich scraps, and finally the fermentation was then statically performed for 96 h at 37 °C. The number of viable bacteria was determined by the plate counting method.

The isolated and purified strains were preliminarily identified by colony morphological observation, gram staining, and physicochemical identification. The shape, size, surface, shape, and color, of the colonies were recorded [23]. The bacterial solution was diluted to 2 McFarland standard turbidity with 0.85% normal saline, and 1-2 drops of each sample were added to each microbial, and incubated at 37 °C for 24 h. According to the instructions of the LAB biochemical identification kit (Guangdong Huankai Microbial Technology Co., LTD, Guangdong, China), we observed the color change to determine whether it was *Lactobacillus*.

The screened strains were inoculated in MRS liquid medium with 1% inoculation amount, cultured overnight at 37 °C, centrifuged at 16,960× *g* for 2 min, and the supernatant was discarded. The genome of the target strain was obtained according to the requirements of the bacterial genome rapid extraction kit (Vazyme Biotech Co., LTD, Nanjing, China). The universal primers of bacteria were selected for PCR amplification, and the reaction procedure was as follows: 94 °C pre-denaturation for 5 min; 94 °C denaturation for 30 s; 54 °C annealing for 30 s; 72 °C extension for 90 s and 35 cycles; 72 °C final extension for 5 min. The target gene fragment obtained by PCR amplification was sent to Sangon Bioengineering (Shanghai, China) Co., LTD for 16S rRNA sequencing. The sequences were submitted to GenBank for alignment, and then the phylogenetic tree was constructed by the MEGA 7.0 software.

#### 2.1.1. Determination of Growth Value and pH Value of Strains

The preserved *L. plantarum* MMB-05 was inoculated in 10 mL MRS liquid medium. It was incubated at 37 °C and inoculated in fresh MRS liquid medium with 1% inoculation for 72 h, and sampled regularly. The OD value was measured at 600 nm, and pH value of the strain was recorded.

#### 2.1.2. Salt Tolerance Test and Antibacterial Activity of *L. plantarum* MMB-05

The activated *L. plantarum* MMB-05 was inoculated into MRS liquid medium (50 mL) containing 0, 2, 4, 6, and 8 g NaCl with 1% inoculation. The OD value were observed at 600 nm for 4, 8, 12, 24, 36, 48, 60, and 72 h [24].

The antibacterial activity was determined by well diffusion agar method. *Escherichia coli* CICC 10,003, *Staphylococcus aureus* CICC 23,656, and *Bacillus cereus* CICC 23,828 were used as indicator bacteria. The isolated *L. plantarum* MMB-05 was inoculated in MRS liquid medium, cultured at 37 °C for 48 h, centrifuged at 11,860× *g* for 15 min, then the supernatant was taken and stored at 4 °C for later use. The indicated bacteria were inoculated in the solid medium with 1% inoculation, drilled with a sterile punch, and 50 µL supernatant was added to each hole. After incubation at 37 °C for 12-16 h, the size of the inhibition zone was measured. The diameter (mm) of the inhibition zone was measured with a vernier caliper, indicating the antibacterial activity of the fermentation broth.

### 2.2. Preparation of P. yezoensis Sauces and Fermentation

The preserved *L. plantarum* MMB-05 and *L. casei* FJAT-7928 were inoculated into MRS liquid medium in glycerol tube, and after two subliminations the culture reached logarithmic stage. Meanwhile, the mixed bacteria (1:1, *v*/*v*) and the control group (without lactic acid bacteria) were set to perform the fermentation under the same conditions. We accurately weighed 100 g of dried seaweed sandwich and added 150 mL of distilled water to form a paste. The mixture was autoclaved at 121 °C for 30 min. After cooling to room temperature, 7.5 mL of bacterial solution was added to the *P. yezoensis* sauce for fermentation [22]. The fermentation temperature was set at 37 °C, and the static fermentation time was 0, 12, 24, 48, and 72 h, respectively.

#### 2.2.1. Extraction of Supernatant

The supernatant of *P. yezoensis* sauce was extracted by the water extraction method after fermentation [25]. The sample was accurately weighed, diluted, and mixed with 50 mL deionized water. The magnetic stirrer was stirred for 30 min and centrifuged at 14,130× *g* for 15 min. This process was repeated twice under the same conditions, and the precipitation was discarded to obtain the supernatant.

#### 2.2.2. Sensory Evaluation

Sensory evaluation was used to compare the differences in various fermented *P. yezoensis* sauces, which was performed in standard sensory laboratories. The four groups of *P. yezoensis* sauce fermented for 24, 48, and 72 h, were evaluated by 30 public officials (12 women and 18 men, aged 21–25). None of them had a taste disturbance, and each participant had at least six months of sensory experiments. Nose clips were worn throughout the sensory process to prevent potential flavor and odor interactions. Each sensory assessor scored the samples based on their fishy taste, sour taste, bitter taste, aroma, salty taste, color, and overall quality of the samples, on a rating scale of 1–9 points where 1 represents the worst quality, and 9 the highest [26].

#### 2.2.3. Determination of Basic Physicochemical Indexes

A digital viscometer (NDJ-5S, Shanghai Fangrui Instrument Co., LTD, Shanghai, China) was used for viscosity measurement. Approximately 100 g *P. yezoensis* sauce was taken and stir with a rotor at a speed of 60 r/min for 20 min. A water activity meter (HD-6, Wuxi Huako Instrument Co., LTD, Wuxi, China) was used to measure the water activity (a_w_) of the *P. yezoensis* sauce. The color difference of the *P. yezoensis* sauce was determined by a color meter (Shanghai Yi Electrophysical Optical Instrument Co., LTD, Shanghai, China).

The total viable counts were tested according to a previous procedure described by Du et al. [23]. The cell counts were expressed as colony numbers (Log CFU/mL). The precision pH meter (PHS-3C, Lei Ci Instrument Factory, Shanghai, China) was used for measurement. Briefly, 5 g of *P. yezoensis* sauce was taken, diluted in 10 mL of distilled water, and then measured after mixing. The total sugar content was determined according to GB/T9695.31-2008, and the content was expressed as glucose equivalent.

#### 2.2.4. Total Phenolic and Total Flavonoids Contents

The total phenolic content was determined by a modified Folin-Ciocalteu method, according to the procedure reported by Sowmya et al. [27]. Take 1 mL of the fermentation supernatant into a test tube, add 1 mL of folin phenol reagent and mix thoroughly, allow to stand for 5 min. Then, add 2 mL of 7.5% Na_2_CO_3_ solution, let this stand for 5 min, take a water bath at 45 °C for 10 min, and the absorbance was then measured at a wavelength of 765 nm. The total phenol content was expressed as the equivalent (mg GEA/g DW) of gallic acid per mL of fermentation supernatant.

The content of total flavonoids was assessed spectrophotometrically according to the method proposed by Kim et al. with a slight modification [28]. Two mL of fermentation supernatant was taken in a test tube and neutralized by 0.6 mL of 10% AlCl_3_, then it was mixed and allowed to stand for 6 min. 4 mL of NaOH solution was absorbed and allowed to stand for 15 min. The absorbance values were measured at the wavelength of 510 nm with rutin as the standard. The total flavonoids content was expressed as equivalent (mg RE/g DW) of rutin per mL of fermentation supernatant.

#### 2.2.5. Determination of Organic acid Content and Antioxidant Activities

The organic acid profile was analyzed on the Shimadzu LC-2010A system (Shimadzu, Tokyo, Japan) following a previously described method, with minor modifications [29]. The chromatographic column was Agilent TC-C18 (4.6 × 25 mm, 5 μm) with the detection wavelength of 210 nm. Isogradient elution was performed on 0.08 M KH_2_PO_4_ solution (pH 2.5). The column temperature, flow rate, and injection volume, were 30 °C, 0.7 mL/min and 20 μL.

Total antioxidant capacity (T-AOC determination Kit, A015-1 Colorimetric, Nanjing Jiancheng Technology Co., LTD, Nanjing, China), ABTS free radical scavenging capacity, and FRAP iron ion reducing capacity (Shanghai Yuanye Biotechnology Co., LTD, Shanghai, China), were determined by referring to the purchase kit.

#### 2.2.6. Determination of Free Amino Acid Content

Free amino acids were quantified according to the method by Adeyeye et al. [30] with minor modification. The *P. yezoensis* sauce (2.0 g) was homogenized for 5 min (3 × 1000 rpm) with 15 mL of hydrochloric acid (0.1 M) and centrifuged at 14,130× *g* for 10 min after standing for half an hour. This centrifugation process was repeated twice. Then, the supernatant was combined to a constant volume of 25 mL. Take 10 mL of the solution and add 10 mL of 10% (*w/v*) trichloroacetic acid (TCA), allow to stand for 1 h and then centrifuge it at 14,130× *g* for 10 min at 4 °C. The supernatant was adjusted to pH 2.0 with 6 M NaOH and 1 M NaOH. Finally, the content was determined using an amino acid analyzer after a 0.22 μm aqueous filter membrane. The analytical conditions were as follows: analytical column, 2622PH 4.6 mm I.D. × 60 mm; flow rate, 0.40 mL/min; column temperature, 57 °C; reaction temperature, 135 °C; detection wavelength, 570 nm; injection volume, 20 μL.

#### 2.2.7. Determination of Volatile Substances by GC-IMS

The determination of volatile substances was done according to the method previously established, with minor modifications [31,32]. Briefly, the accurately weighed 2.0 ± 0.1 g of *P. yezoensis* sauce and placed in a 20 mL headspace bottle. The vial was then kept in a water bath for 20 min at 80 °C, after which samples were injected. The analytical conditions were as follows: column, wax, 30 m; ID, 0.53 mm; film thickness, 1 μm (RESTEK, USA); column temperature, 60 °C; carrier gas, N_2_; IMS temperature, 45 °C; injection volume, 500 μL; injection needle temperature, 85 °C; incubation speed, 500 rpm; analysis time, 40 min; drift gas, 150 mL/min.

### 2.3. Statistical Analysis

All analyses were performed in triplicate and data is presented as means ± standard deviations. One-way analysis of variance (ANOVA) with Tukey method and significant difference expressed at *p* < 0.05 level. VOCal, a qualitative and quantitative analysis software, was used for the identification of compounds, and the NIST database and the IMS database were used for qualitative analysis of volatile substances. The spectral differences and fingerprints were compared by Gallery Plot and Reporter plug-in and volatile substances of four fermentation samples were analyzed by dynamic principal component analysis (PCA). Excel 2010, SPSS statistics 26 and Origin 2021 were used for data processing and drawing.

## 3. Results and Discussion

### 3.1. Identification of L. plantarum MMB-05

A total of ten presumptive LAB strains were isolated from pickle brine and used for the fermentation of sandwich seaweed scraps. The results showed that all selected strains were grow in *P. yezoensis* sauce, and the number of viable cells showed a trend of increasing first and then decreasing during fermentation (Table S1). However, after 96 h of fermentation, the number of colonies of MMB-05 remained at 11.43 ± 0.08 CFU/mL. Therefore, the strain of MMB-05 was selected for the further study.

The physiological and biochemical identification was carried out according to the methods in the literature [33]. The colonies were milky white, opaque, with regular edges and smooth surfaces. Microscopic examination showed that a single strain was straight, or curved short rod, or chain, with positive gram stain (Figure S1). According to Table 1, it could be concluded that the target strain accords with the characteristics of lactic acid bacteria. A comparison of the developmental trees established by BLAST procedure showed that the sequence similarity between this strain and *L. plantarum* was 100%, and confirmed that the screened strain was *L. plantarum* (Figure S2). Therefore, the bacterium was named as *L. plantarum* MMB-05.

### 3.2. Growth Characteristics

**Figure 1:**
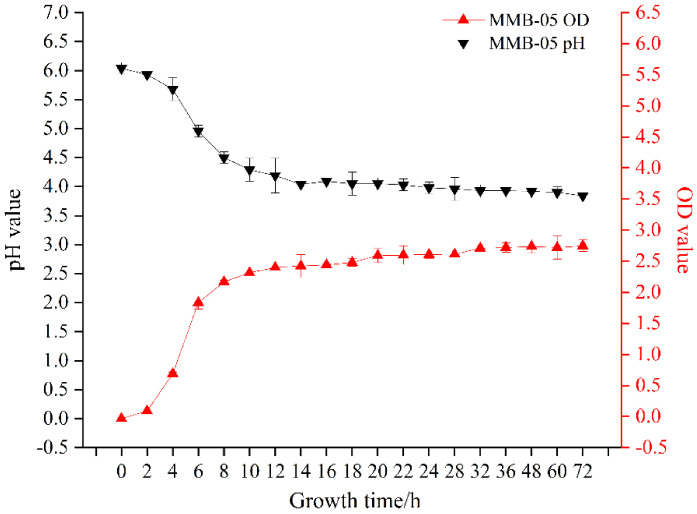
Growth and pH changes in *L. plantarum* MMB-05.

The growth characteristics of *L. plantarum* MMB-05 was shown in Figure 1, *L. plantarum* MMB-05 entered the logarithmic phase from 2 to 8 h, and then entered the stable phase. At the same time, the pH value of *L. plantarum* MMB-05 decreased from 6.04 ± 0.10 to 3.84 ± 0.00 and entered a rapid decline stage from the 4th hour, which showed a good capacity for acid production. Obviously, the pH value was negatively correlated with the growth value. The decrease in pH is associated with the accumulation of metabolite lactic acid [34].

**Figure 2:**
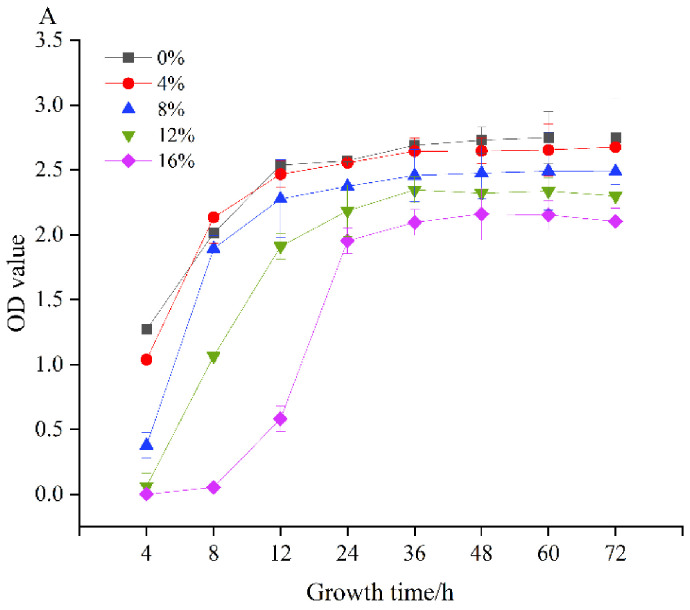

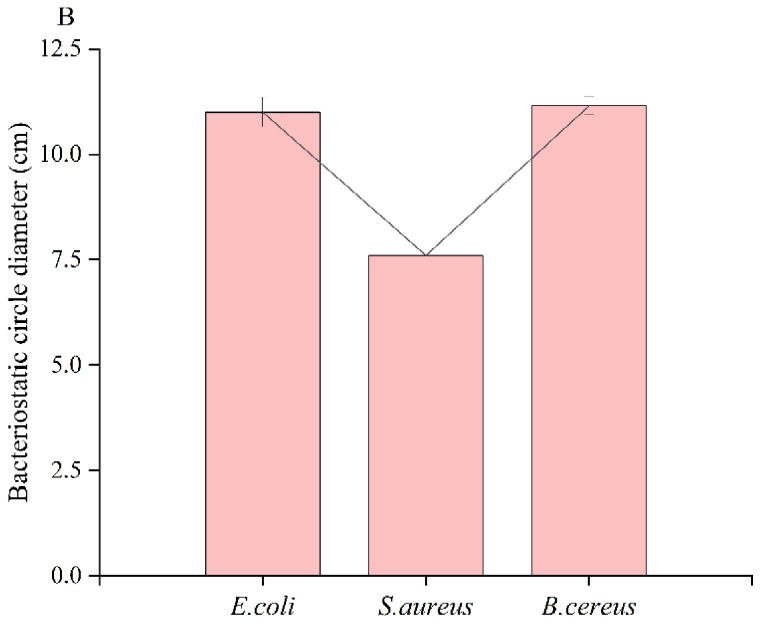
Salt tolerance (**A**) and antibacterial activity (**B**) of *L. plantarum* MMB-05.

Figure 2A shows the growth of *L. plantarum* MMB-05 at different NaCl concentrations. There was certain growth under different salt concentrations, and the growth value decreased significantly with the increase of salt content (*p* < 0.05). It is worth noting that all concentrations showed a trend of rising first and then stabilizing. The strain still grew at 16% NaCl concentration. This is in line with the study conducted by Hiroshi et al. [35], which showed that strains could grow to a certain degree at 0-15% concentrations. In addition, *L. plantarum* MMB-05 had significant antibacterial effect against *E. coli*, *S. aureus* and *B. cereus*, with diameters of 11.00 ± 0.35, 7.60 ± 0.14 and 11.15 ± 0.21 cm, respectively, which was generally consistent with the results of relevant studies (Figure 2B). Cheong et al. [36] found that 12 strains of *L. plantarum* had fungicidal activity. Zangeneh et al. [37] concluded that the supernatant of *L. plantarum* had a significant inhibitory effect on pathogenic bacteria, especially *S. aureus* and *E. coli*.

### 3.3. Sensory Evaluation

**Figure 3:**
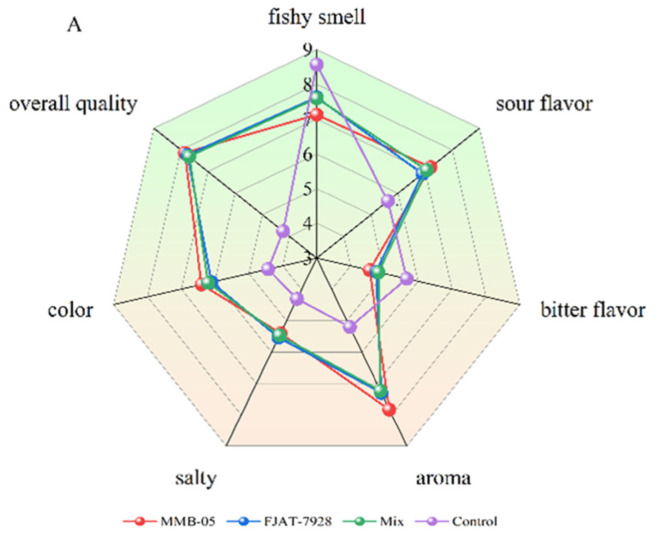

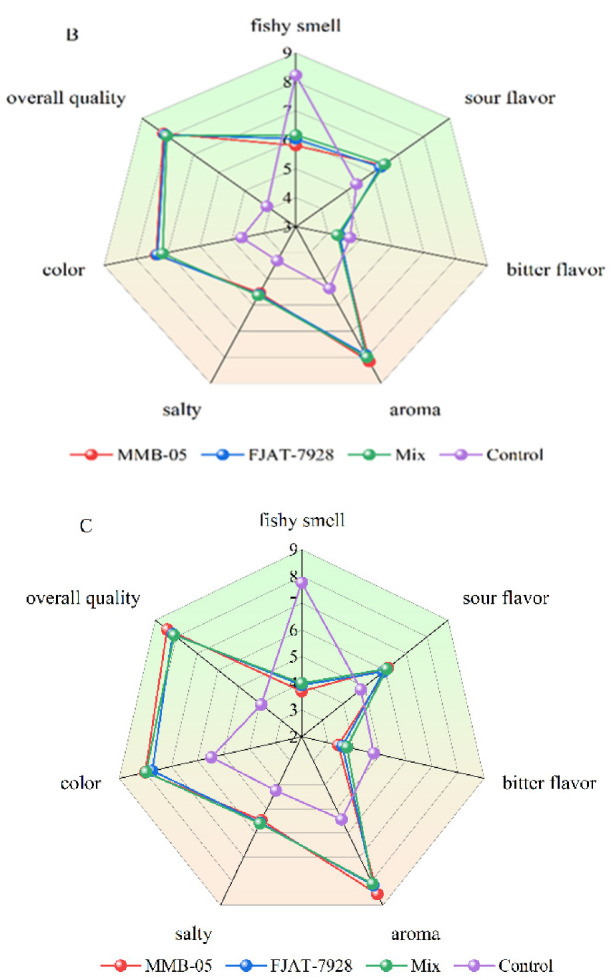
Sensory evaluation radars of *Porphyra yezoensis* sauce fermented for 24 (**A**), 48 (**B**) and 72 (**C**) h, respectively.

The sensory evaluation results of fermented *P. yezoensis* sauce are presented in Figure 3. Regardless of what kind of LAB starter was used, the aroma, color, and overall quality of the final product were very high, indicating that the fermented *P. yezoensis* sauce was acceptable to consumers. However, the overall quality of the control group was much less than that of the lactic acid bacteria fermentation group. At 24 h of fermentation, the fish and sour taste of the lactic acid bacteria fermentation group were higher. At the end of the fermentation, the bitter flavor, fishy smell, and sour flavor decreased, with the fishy smell in *L. plantarum* MMB-05 fermentation group being the lowest, followed by *L. casei* FJAT-7928 and mix fermentation groups. Compared with the control group, the *P. yezoensis* sauce fermented by lactic acid bacteria had little difference in salty taste, but the aroma was better than that of the control group. In terms of the overall quality, *L. plantarum* MMB-05 fermentation group scored the highest, followed by *L. casei* FJAT-7928, mixed fermentation, and control fermentation groups. All the flavor and odor changes may be due to the production of different volatile compounds during lactic acid bacteria fermentation. Overall, the edible properties of *P. yezoensis* sauce was significantly improved, and the texture and sensory properties of *P. yezoensis* sauce were enhanced after the fermentation of lactic acid bacteria.

### 3.4. Physicochemical Indexes

A_w_ is one of the most important factors affecting fungal growth. The a_w_ values of *P. yezoensis* sauce fermented by lactic acid bacteria decreased significantly (*p* < 0.05) over the course of this study (Table 2). The a_w_ value of all groups reached about 0.75 after 72 h of fermentation. Among them, the a_w_ value of *P. yezoensis* sauce fermented by *L. plantarum* MMB-05 was the lowest, while the a_w_ value of control group was 0.81. After 72 h, the a_w_ values of the four groups varied significantly (*p* < 0.05). All the above results confirmed that the a_w_ value of the samples could be reduced to below 0.80 by the fermentation of the three lactic acids bacteria. The reduction of water activity could reduce the water available to microorganisms, which could help improve the safety of products and inhibit the growth of miscellaneous bacteria [38]. Therefore, inoculation of lactic acid bacteria to ferment *P. yezoensis* sauce can significantly improve the preservation and safety of the product.

As a visual quality characteristic, color is an influential parameter of food acceptability for consumers. At the end of fermentation, the L* values of the four groups were all in the range of 25–29 (Table 2), which was in accordance with the results for sandwich nori [39]. With the prolongation of fermentation time, the viscosity value of all groups decreased significantly (*p* < 0.05) (Table 2). Meanwhile, the viscosity value of *P. yezoensis* sauce also showed significant differences between the different groups. The viscosity values of *L. plantarum* MMB-05, *L. casei* FJAT-7928, and the mix fermentation groups, decreased by 52.84%, 30.39%, and 57.69% after 72 h of fermentation, while the viscosity value of the control group was 494.69 cp, possibly due to the fermentation of sugars and proteins by lactic acid bacteria producing a large number of free amino acids and organic acids.

### 3.5. Viable Cell Counts, pH, and Total Sugar

**Figure 4:**
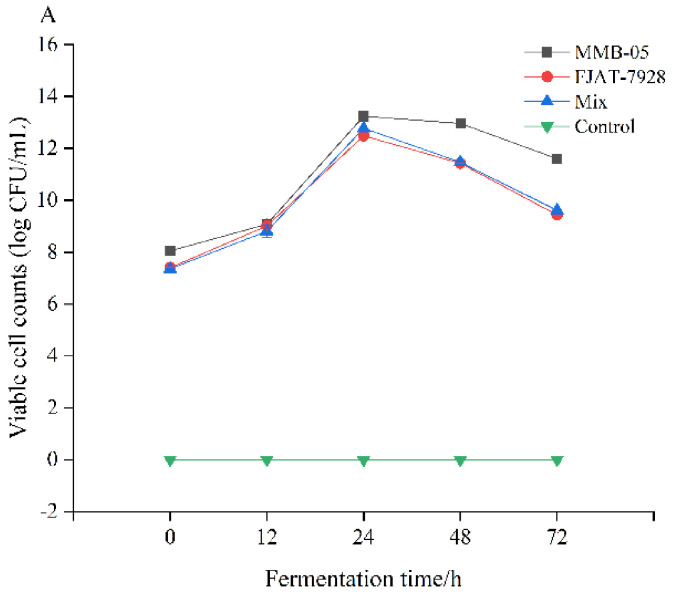

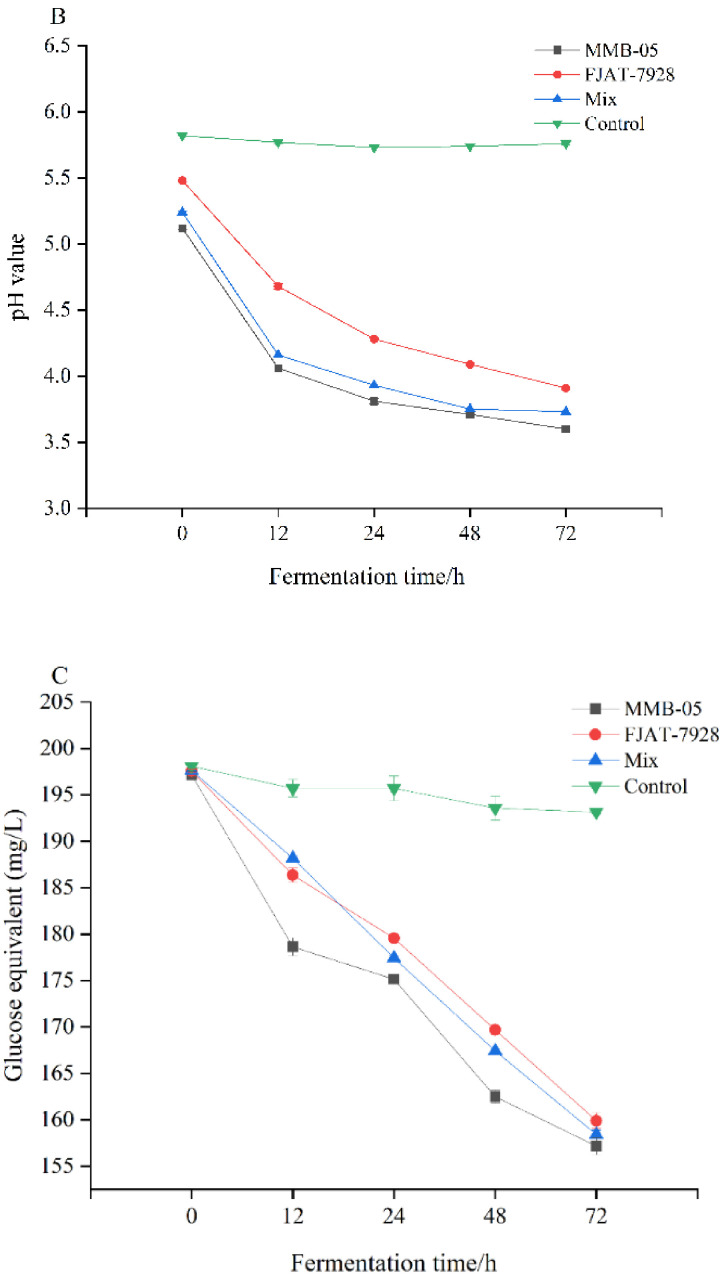
Changes in viable cell counts (**A**), pH value (**B**), and total sugar content (**C**), during 3 days LAB stains fermentation of *P. yezoensis* sauce.

Changes in viable cell counts, pH, and the total sugar content of the four groups of *P. yezoensis* sauce are shown in Figure 4. All strains could grow normally in the sauce after inoculation, and the count of viable cell increased first and then decreased (Figure 4A). During the fermentation period, the count of viable cell in the *L. plantarum* MMB-05 fermentation group was consistently higher than that in the other groups, especially after 24 h (up to 13.24 ± 0.01 log CFU/mL). During the fermentation period of 0 to 24 h, the count of viable cell in the *L. casei* FJAT-7928 fermentation group was slightly higher than the mix fermentation group, and the opposite trend occurred after 24 h of fermentation. The count of viable cell after 72 h fermentation was always higher than the number after initial inoculation, indicating that all three strains could grow in the *P. yezoensis* sauce for a long time, and the growth status of *L. plantarum* MMB-05 was more prominent.

The pH value of the *P. yezoensis* sauce in each group showed a continuous downward trend, which decreased significantly during the 0–24 h, and was negatively correlated with the number of viable bacteria (Figure 4B). In the early stage of fermentation, the rapid reduction of pH value could effectively inhibit the reproduction of other acid-intolerant bacteria, thereby reducing the accumulation of harmful metabolites. The initial pH values of *L. plantarum*, *L. casei*, mix and control groups, were 5.12 ± 0.01, 5.48 ± 0.01, 5.24 ± 0.01, and 5.82 ± 0.01, respectively, which dropped sharply to 3.6 ± 0.01, 3.91 ± 0.01, 3.73 ± 0.02, and 5.76 ± 0.01, at the end of fermentation. The pH of other fermenting bacteria has been reported to vary from 3.3 to 4.6 at the end of the fermentation, depending on the fermentation temperature, the amount of carbohydrates available, or the additives used during the fermentation [40].

Microorganisms consume carbohydrates for their metabolic activities during fermentation. Figure 4C shows the total sugar content in the fermented *P. yezoensis* sauce with different strains. At the end of the fermentation, the total sugar content of the control group was 193.16 ± 0.19 mg/L. Compared with the other two lactic acid bacteria fermentation groups, *L. plantarum* MMB-05 had the greatest effect on the total sugar content of *P. yezoensis* sauce, and its total sugar content decreased by 20.29% after fermentation. The results showed that the strain consumed more carbohydrate than other strains, and the reduction of carbohydrate during fermentation was associated with the conversion of lactic acid and the metabolism, growth, and reproduction of *L. plantarum* [41]. This also explains the higher number of viable bacteria in the *P. yezoensis* sauces inoculated with *L. plantarum* MMB-05.

### 3.6. Total Phenolic, Flavonoids Contents and Organic Acids

**Figure 5:**
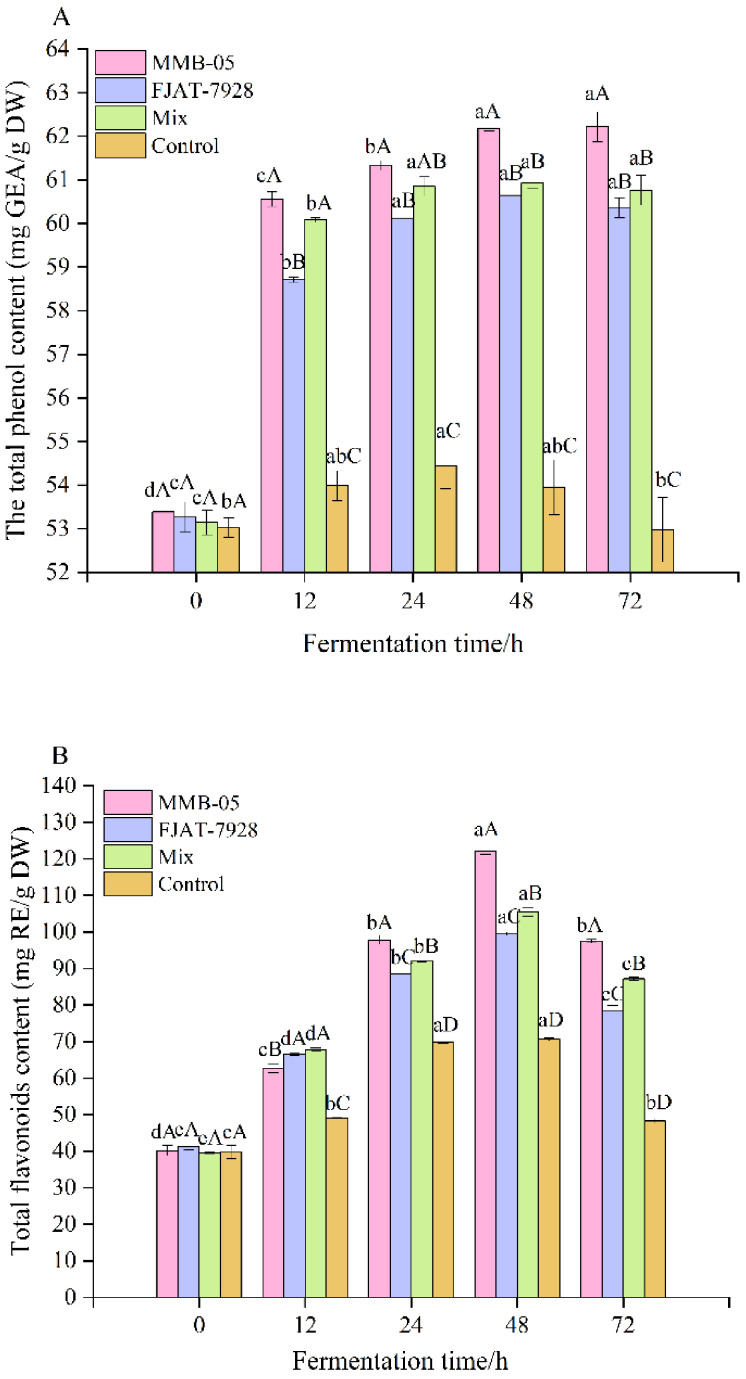
Changes in total phenol (**A**) and total flavonoids (**B**) contents during LAB stains fermentation of *P. yezoensis* sauce. Note: abcd represents the significant difference between groups (different fermentation time in the same group). ABCD represents the significant difference between the same fermentation time of different groups (*p* < 0.05).

The total phenolic content of the three lactic acid bacteria fermentation groups continued to increase throughout the fermentation process (Figure 5A). After 12 h of fermentation, the total phenolic content of *P. yezoensis* sauce fermented by three lactic acid bacteria was significantly higher than that in the control group. Moreover, the total phenolic content of *L. plantarum* MMB-05 fermentation group was significantly higher than the other groups (*p* < 0.05), a 16.54% increase than the initial content after 12 h of fermentation. These results indicate that *L. plantarum* fermentation could significantly increase the total phenol content of the fermentation supernatant of *P. yezoensis* sauce. This was in line with the increase in total phenolic content reported by Di Cagno et al. [42] and Kwaw et al. [43] during fermentation. These increases in the total phenol content could be related to the release of corresponding glycoside element by microbial enzymes, such as decarboxylase, reductase, and tannase [44,45].

With the increase of fermentation time, the total flavonoids content in the four groups showed a trend to increase first and then decrease (Figure 5B). During the first 48 h of fermentation, the total flavonoids content in the supernatant of the *L. plantarum* MMB-05, *L. casei* FJAT-7928 and mix groups increased from 41.17, 41.33, and 39.50 mg/mL to 122.17, 99.50, and 105.50 mg/mL (*p* < 0.05), respectively. The content of total flavonoids in the control group was significantly lower than that in the other three groups. It was found that different kinds of *lactobacillus* species had different ability to release total flavonoids by fermentation. The increase in total flavonoids content is related to changes in the acid level in the fermentation environment, which release the combined flavonoid compounds. Another reason for the increase in flavonoid content is that microorganisms could secrete enzymes such as *β*-glucosidase during fermentation, thereby releasing more flavonoids [46].

**Figure 6:**
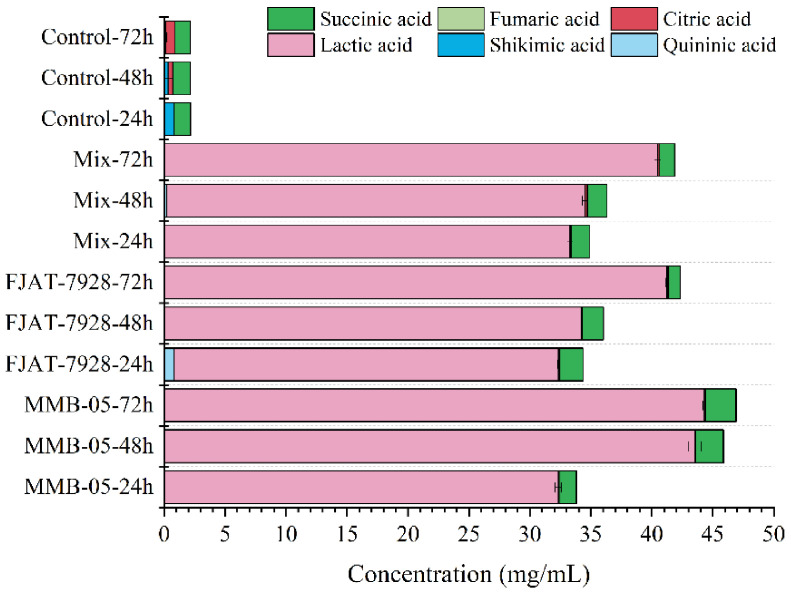
Changes in organic acid contents during LAB stains fermentation of *P. yezoensis* sauce.

Organic acids are important components that affect sensory and chemical properties of food products as well as microbial stability [47]. Changes in organic acid content during fermentation were examined in this study (Figure 6). A total of six organic acids were detected, and lactic acid was produced in the *P. yezoensis* sauce fermented by lactic acid bacteria during fermentation, compared with the control group. At the same time, the content of succinic acid in *P. yezoensis* sauce fermented by *L. plantarum* MMB-05 increased by 74.69%, and the content of succinic acid in *P. yezoensis* sauce fermented by *L. casei* FJAT-7928 and the mix groups decreased. Other organic acids were low in content and therefore have little effect on the flavor of *P. yezoensis* sauce. The kind and content of organic acids in *P. yezoensis* sauce fermented by different lactic acid bacteria were different. After fermentation by lactic acid bacteria, the saccharide content in supernatant decreased obviously due to the conversion of some saccharide substances into organic acids. Overall, the total organic acid content of the three lactic acid bacteria fermentation groups gradually increased as the fermentation time increased.

### 3.7. In Vitro Antioxidant Capacity

**Figure 7:**
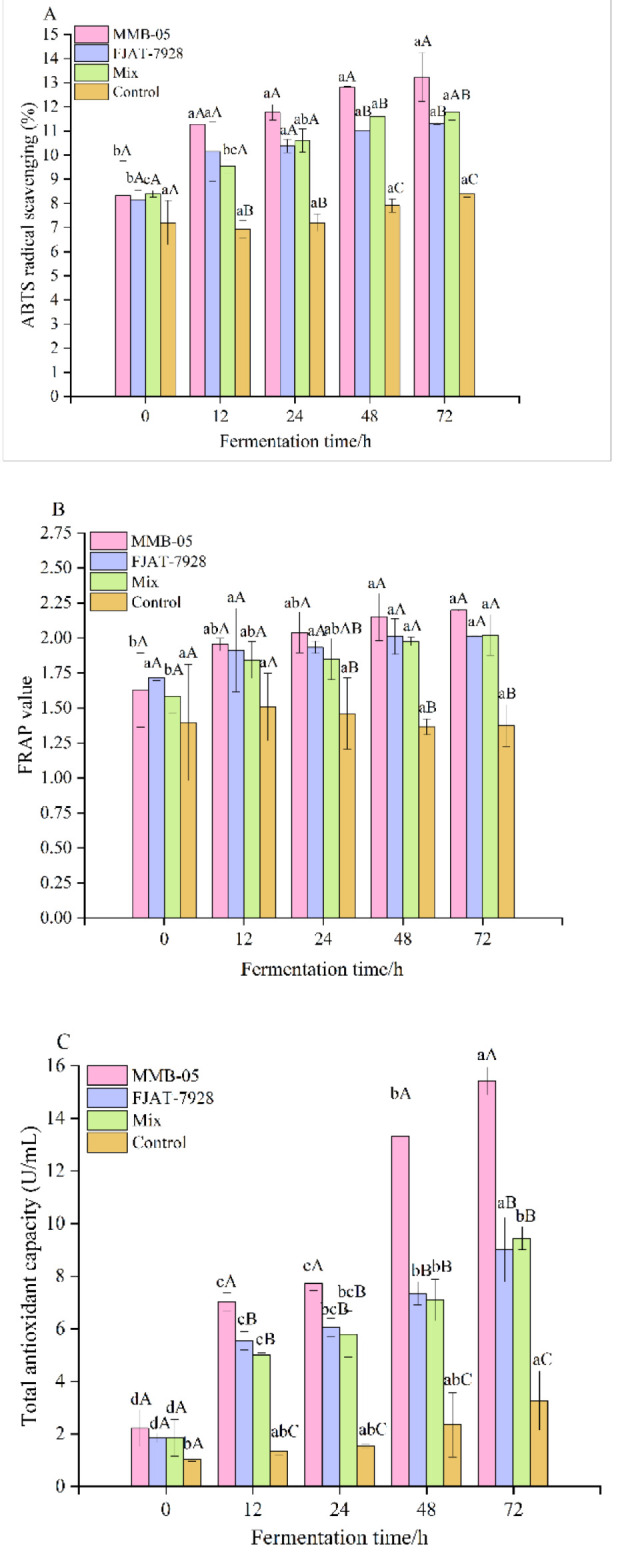
ABTS^+^ radical scavenging (**A**), FRAP (**B**) and total antioxidant capacity (**C**), in *P. yezoensis* sauce fermented by LAB stains. Note: abcd represents the significant difference between groups (different fermentation time in the same group). ABC represents the significant difference between the same fermentation time of different groups (*p* < 0.05).

Three systems (ABTS^+^ radical scavenging capacity, FRAP, and total antioxidant capacity) were selected in our study to evaluate the antioxidant activity the supernatants of four *P. yezoensis* sauces. Figure 7A shows that the ABTS scavenging ability of different lactic acid bacteria strains increased with the increase of fermentation time. Among them, the scavenging ability of *L. plantarum* MMB-05 fermentation group was relatively high, which increased from 8.33 to 13.23%, whereas the ABTS scavenging ability did not change significantly in the control group.

The supernatants of the four *P. yezoensis* sauces showed varying degrees of reducing activity against the iron ions (Figure 7B), which increased with the fermentation time. During fermentation, the reducing activity of *L. plantarum* MMB-05 was significantly higher than that of the other three samples. At the end of fermentation, the maximum FRAP value was achieved by the three lactic acid bacteria fermentation groups. Similarly, Chen et al. [48] also found that the scavenging activity of ABTS^+^ and could be enhanced when *Eurotium cristatum* YL-1 was used for solid-state fermentation in soybeans.

In addition, the total antioxidant capacity of the three lactic acid bacteria fermentation groups increased significantly after 12 h of fermentation (*p* < 0.05) (Figure 7C). During the fermentation process from 48 to 72 h, the total antioxidant capacity of *L. plantarum* MMB-05 group increased significantly from 2.22 ± 0.70 to 15.42 ± 0.52 U/mL. However, the total antioxidant capacity of *L. casei* FJAT-7928 and mix fermentation groups did not increase significantly. Meanwhile, the total antioxidant capacity of the control group remained unchanged. Antioxidant activity was associated with the presence of total phenols, which is why the results showed a similar increasing trend. It is undeniable that the improvement of antioxidant activity is related to the growth and metabolism of thallus, which may release some bioactive peptides [49].

### 3.8. Free Amino Acids

The accumulation of free amino acids during the fermentation process could not only improve the taste of the samples, but also the α-keto acids produced by microbial metabolism could enter the tricarboxylic acid cycle and promote the formation of flavor substances. According to the classification of amino acids taste by Kato et al. [50] and Schoenberger et al. [51], free amino acids were divided into umami amino acids (Asp and Glu), sweet amino acids (Asp, Thr, Ser, Gly, Ala, Val, Phe, and Pro), bitter amino acids (Val, Met, Ile, Leu, Tyr, Phe, Lys, His, Pro, and Arg), and sour amino acids (Glu and His).

Changes in free amino acid contents during fermentation were compared and shown in Table 3. Among the 17 kinds of free amino acids, except the control group, the content of free amino acids increased significantly with the fermentation in the other three groups (*p* < 0.05). The contents of umami, sweet, sour, and bitter amino acids, in the other three fermentation groups, increased with time. The initial contents of sweet amino acids in *L. plantarum* MMB-05, *L. casei* FJAT-7928 and the mix fermentation groups were 4.5 ± 0.01, 3.37 ± 0.04, and 4.13 ± 0.01 mg/g, respectively, which increased to 6.14 ± 0.02, 5.81 ± 0.01, and 5.92 ± 0.02 mg/g at the end of fermentation. At the end of fermentation, the contents of umami amino acids, sour amino acids, bitter amino acids, and total free amino acids, in the *L. plantarum* MMB-05 fermentation group increased by 39.93%, 35.84%, 32.31%, and 36.33%, respectively. The bitter amino acids content of the *L. casei* FJAT-7928 fermentation group increased by 64.15%, and the bitter amino acids content of the mix fermentation group increased by 45.98%. It is worth noting that glutamic acid, alanine, and histidine, accounted for more than 50% of the total free amino acids content. During fermentation, the increase in free amino acids content is mainly due to the hydrolysis of proteases and peptidases [52]. However, the contents of umami amino acids and sour amino acids decreased gradually in the control group, while the opposite occurred with the bitter amino acids, and the content of sweet amino acids showed a trend of decreasing first and then rising, resulting in a slightly worse overall flavor of the *P. yezoensis* sauce.

**Figure 8:**
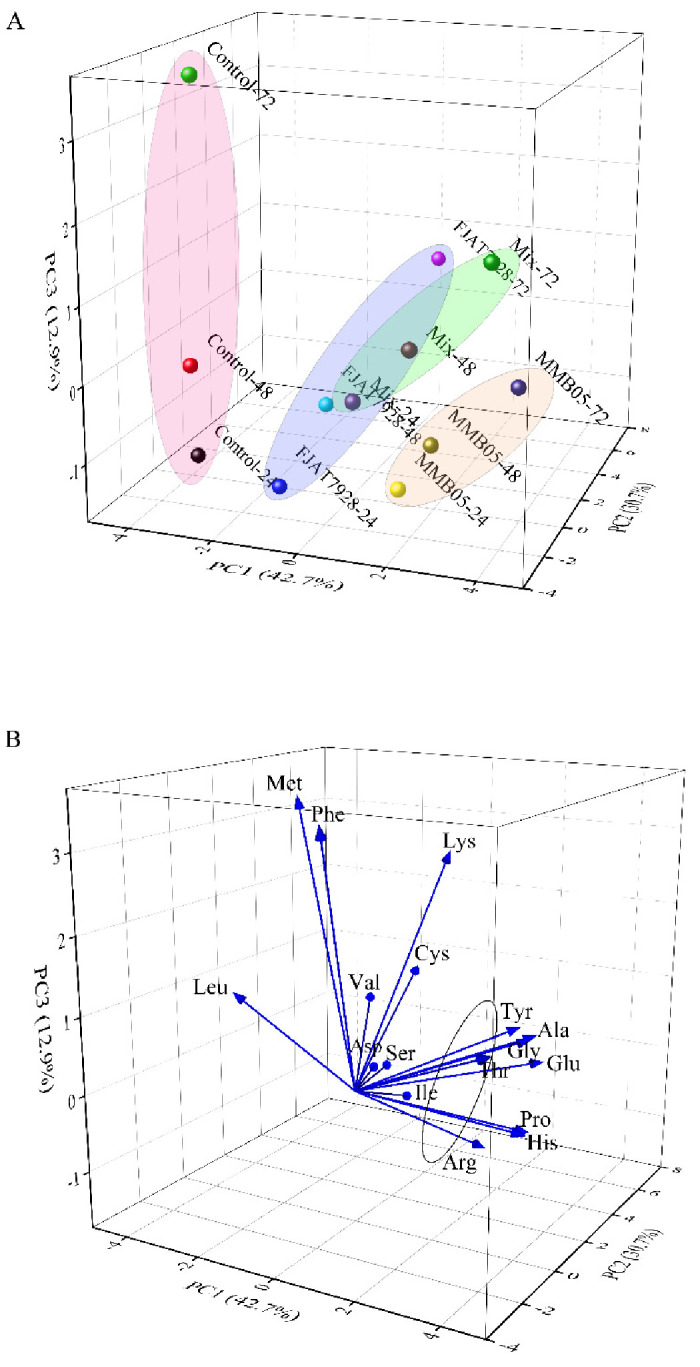
Principal component diagram of free amino acid content changes during LAB stains fermentation of *P. yezoensis* sauce. score plot (**A**); loading plot (**B**).

The principal component analysis of free amino acids in *P. yezoensis* sauce was carried out (Figure 8A), and three principal components were obtained, accounting for 41.5%, 30.7%, and 12.9%, respectively. The PC1 values of the three lactic acid bacteria fermentation groups increased with the fermentation, and the close distance between the *L. plantarum* MMB-05 and the mix fermentation groups indicated that the changes in free amino acid contents were similar in both groups. However, the values of PC1 and PC2 in the control group did not change significantly with the fermentation. As described in Figure 8B, lactic acid fermentation resulted in higher PC1 values for Pro, Ala, Thr, Gly, and His, especially during the later fermentation stages. From the overall flavor analysis, the flavor and nutrition of free amino acids were more prominent in the *L. plantarum* MMB-05 fermentation group.

### 3.9. Volatiles Compounds Analysis

The volatile compounds of *P. yezoensis* sauce fermented for 72 h were both qualitatively and quantitatively analyzed by GC-IMS. A total of 109 volatile compounds were detected, including 34 aldehydes, 24 alcohols, 16 ketones, 8 esters, 4 acids, 2 aromatic hydrocarbons, 6 furans, 5 pyrazines, 1 pyridine, and 9 other compounds.

**Figure 9:**
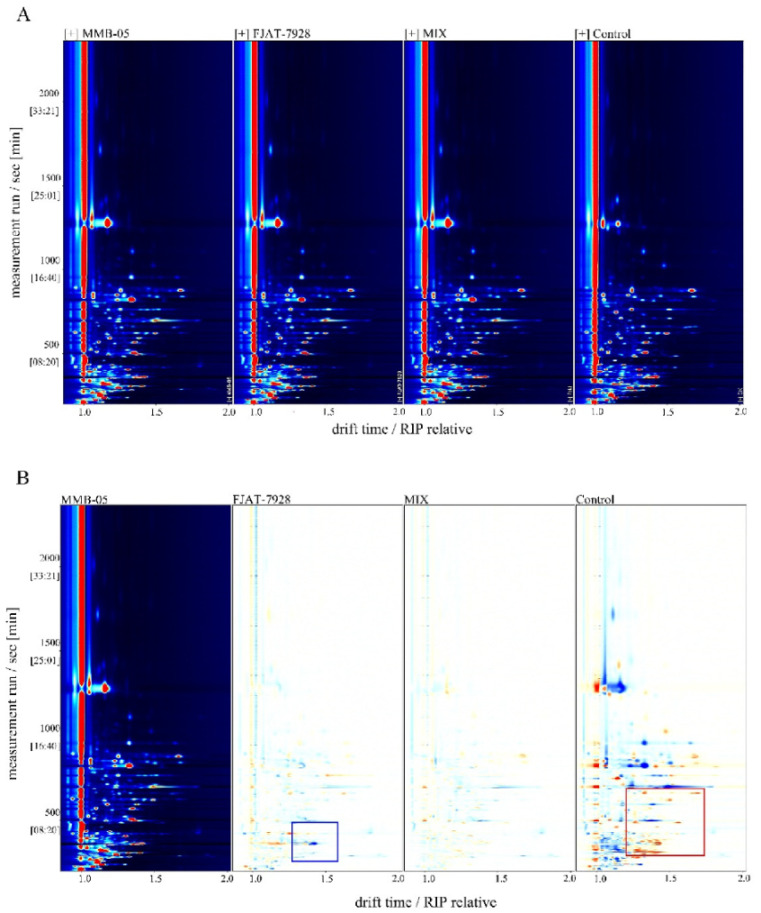

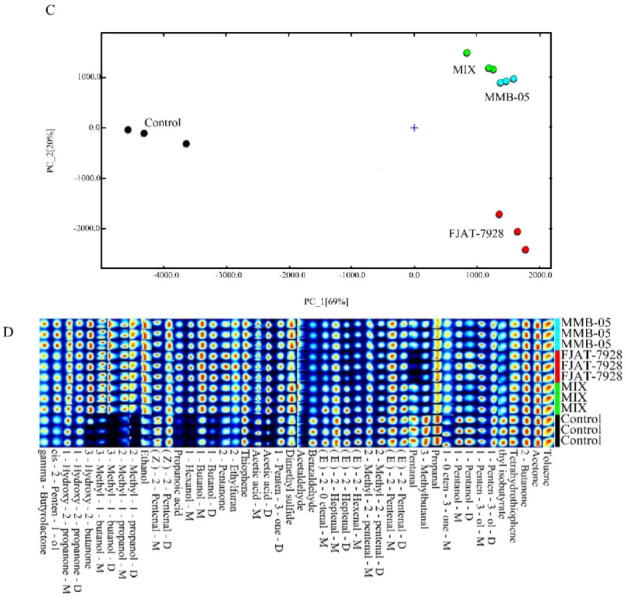
The volatile organic compounds analysis of 72 h fermented *P. yezoensis* sauce by GC-IMS. 3D-topographic top view plot (**A**); difference map of 3D-topographic top view plot (**B**); PCA score chart (**C**); fingerprint spectrum of some of the volatile organic compounds (**D**).

The GC-IMS 3D topography overlook diagram was displayed in Figure 9A. The red vertical line at abscissa 1.0 is the RIP peak (reaction ion peak, normalized). The vertical axis represents the GC retention time (s) and the horizontal axis represents the ion migration time (normalized). Each point flanking the RIP peak represents a volatile organic compound, mainly occurring during a migration time of 1.0–1.75 and a retention period of 300–1500 s. The color represents the concentration of the substance, with white indicating a lower concentration and red indicating a higher concentration; the darker the color, the higher the concentration. The signal peaks in *L. plantarum* MMB-05 were deducted from *L. plantarum* MMB-05 as a reference to obtain the differential spectra (Figure 9B). The blue area indicates that the substance is lower than *L. plantarum* MMB-05 in this sample, while the red area indicates higher than *L.* plantarum MMB-05. The blue area indicates that the substance was lower than *L. plantarum* MMB-05 in this sample, while the red area indicates higher than *L. plantarum* MMB-05. Similarly, the darker the color, the greater the difference. It is necessary to conduct in-depth statistical analysis (Table 4) to integrate the volatility profile information of the different samples.

#### 3.9.1. Principal Component Analysis of Volatiles Compounds

Principal component analysis was used to identify various fermented *P. yezoensis* sauces (Figure 9C). The total contribution rate of the first two principal components was 89%, with PC1 and PC2 accounted for 69% and 20%, respectively, which was sufficient to explain the similarity between the different samples [53]. From the degree of aggregation and dispersion of the samples, it could be seen that the fermentation group of *L. plantarum* MMB-05 was closer to the mix fermentation group, indicating that the flavor composition was similar in both groups, while the fermentation group of *L. casei* FJAT-7928 was far apart, indicating that there was a certain difference in the flavor components of the two groups. This phenomenon is largely consistent with the change of free amino acid content. Studies have shown that different strains of lactic acid bacteria metabolize nutrients depending on their specific transport modes. It is known that *L. plantarum* is facultative hetero-fermentative and *L. casei* is homogenous. Therefore, they produce different types and amounts of metabolites and have different effects on environmental characteristics, such as pH and oxygen availability, which in turn may affect the volatility of fermented samples [54].

#### 3.9.2. Aroma Analysis

For a more intuitive quantitative comparison of the differences in volatile organic compounds between different samples, the compounds with higher content were screened and the fingerprint spectrum was established (46 compounds) (Figure 9D). The contents of acetic acid-M, acetic acid-D, 1-hydroxy-2-propanone-M, 3-hydroxy-2-butanone, (Z)-2-pentenal-D, ethanol, 2-butanone, acetone, gamma-butyrolactone, 3-methyl-3-butanol-D, and other substances in the *L. plantarum* MMB-05, *L. casei* FJAT-7928, and the mix fermentation groups, were relatively higher. At the same time, the contents of phenylacetaldehyde, benzaldehyde, 2-decenal, (E,E)-2,4-octadienal, (E)-2-octenal-M, (E)-2-heptenal-M, nonanal, octanal-M, hexanal-M, pentanal, 3-methylbutanal, butanal, propanal, diethyl acetal, furfural, 1-octen-3-ol, 1-pentanol-M, cyclohexanone, ethyl butanoate-M, ethyl pyruvate, ethyl isobutyrate, alpha-terpinene, alpha-phellandrene, p-xylene, toluene, dimethyl disulfide, and other substances, were higher in the control group. A lager number of aldehydes were abundant in the control group, but their content decreased after fermentation, while the content of alcohols increased in the samples fermented by lactic acid bacteria.

The relative contents of aldehydes, ketones, acids, and alcohols, were higher after fermentation. Aldehydes and ketones contributed significantly to the early fermentation flavor of fermented *P. yezoensis* sauce. In our study, due to the short fermentation time, the flavor of *P. yezoensis* sauce is mainly composed of aldehydes and ketones. Compared with the control group, the contents of aldehydes in the three lactic acid bacteria fermentation groups decreased significantly, with little change in ketones, and significantly higher contents occurred for acids and alcohols compounds.

Aldehydes could be formed by oxidative degradation of polyunsaturated fatty acids under the action of microorganisms, with most volatile compounds having low thresholds values and pleasant smells, such as being grassy, malty, fruity, and cheesey [55]. Among them, phenylacetaldehyde has the fruit and sweet taste, and benzaldehyde has the taste of almond. Nonanal is confirmed to be the main body of fishy smell [56]. The contents of nonanal in the three lactic acid bacteria fermentation groups decreased after fermentation, indicating that the extension of fermentation time could reduce the fishy taste of *P. yezoensis* sauce.

Alcohols are mainly produced by lipid oxidation, amino acids, and carbohydrate metabolism [57]. The high threshold values of alcohols contribute little to the overall flavor of the sauce, but some low threshold value of unsaturated alcohols contribute to the overall flavor of the sauce. For example, the relative content of ethanol in the samples fermented by lactic acid bacteria increased to some extent, resulting in a soft alcohol odor. 1-Octen-3-ol is the key substance that produces fishy smell in laver. After fermentation by *L. plantarum* MMB-05, its relative content decreased significantly, which had a certain impact on the deodorization of *P. yezoensis* sauce.

The relative content of acids increased significantly after fermentation by lactic acid bacteria, which was associated with the acid production of lactic acid bacteria. Acids have a fruity, cheesy, fatty, and rancid odor, and three short-chain acids were identified from the samples in our study, namely acetic acid-M, acetic acid-D, and propionic acid. Acid is the precursor of esters, and the appropriate content of short-chain acid can enrich the flavor of seaweed sauce and make it refreshing and sweet [58,59].

The relative content of ketones did not change significantly after lactic acid bacteria fermentation. Ketones are stable in properties and have a pleasant aroma of fruit and milk. Among them, 3-hydroxy-2-buxy-3-butanone has a milky flavor, and acetone has sweet, fruity, and etherous aroma. The contents of 3-hydroxy-2-buxy-3-butanone and acetone in the three kinds of lactic acid bacteria fermentation groups were much higher than those in the control group, which had a certain effect on the flavor of *P. yezoensis* sauce. 2-heptanone may be associated with deterioration of raw materials and will bring unpleasant off-flavors to the consumer [60].

2-Pentylfuran has soy, fruity, earthy, green, and vegetable-like aromas, making an important contribution to the flavor. Furfural, contributing to a sweet and almond-like aroma, is the most important furans in *P. yezoensis* sauce. Among them, esters, pyrazine, aromatic hydrocarbons, and pyridine compounds, have the characteristics of high threshold and low content, which have little impact on the overall flavor of *P. yezoensis* sauce.

## 4. Conclusions

This study was designed to screen out the effective lactic acid bacteria that can grow and metabolize in *P. yezoensis* sauce, then the fermentation characteristics of these strains in *P. yezoensis* sauce were studied. During the study’s 72 h fermentation period, all these strains showed strong fermentation ability in *P. yezoensis* sauce, with viable cell counts greater than 10 log CFU/mL after 48 h of fermentation. Notably, *L. plantarum* MMB-05 had significantly higher lactic acid, lower pH value, and higher numbers of LAB. Additionally, the increase in lactic acid led to good bioconversion of phenolic substances in *P. yezoensis* sauce and improved the antioxidant capacity of *P. yezoensis* sauce. In terms of sensory evaluation, *L. plantarum* MMB-05 had the highest score, followed by *L. casei* FJAT-7928, the mix and control group. The increase of free amino acids after fermentation sublimates the flavor and nutrition of *P. yezoensis* sauce. The GC-IMS analysis also demonstrated that *L. plantarum* MMB-05 could produce more than 100 volatile compounds, which increased the sour, sweet, and umami taste of *P. yezoensis* sauce, and improved the overall satisfaction. In general, *L. plantarum* had the best fermentation effect, and the overall quality of 72 h fermentation was acceptable to consumers. Therefore, further research on the fermentation mechanism of *L. plantarum* for *P. yezoensis* sauce will lay a foundation for the development of new products of laver sauce.

## Figures and Tables

**Table 1:** Biochemical identification table of *Lactiplantibacillus plantarum* MMB-05.

Names	Result
seven leaf glycosides	+
fiber the second pond	+
maltose	+
mannitol	+
salicin	+
sorbitol	+
sucrose	+
raffinose	+

Note: + means more than 90% strains are positive.

**Table 2:** Basic physical and chemical indexes of four groups of *Porphyra yezoensis* sauce (water activity, chromatic aberration, and viscosity).

Indicators	Fermentation Time/h	MMB-05	FJAT-7928	Mix	Control
Water activity (a_w_)	0	0.76 ± 0.00 ^aB^	0.80 ± 0.00 ^aA^	0.80 ± 0.00 ^bB^	0.80 ± 0.00 ^aA^
	24	0.75 ± 0.00 ^bC^	0.78 ± 0.00 ^bB^	0.78 ± 0.00 ^cC^	0.79 ± 0.00 ^bA^
	72	0.74 ± 0.00 ^bD^	0.75 ± 0.00 ^cC^	0.75 ± 0.00 ^aB^	0.81 ± 0.00 ^aA^
Chromatic aberration (L*)	0	23.87 ± 0.32 ^bC^	28.39 ± 0.08 ^bA^	28.53 ± 0.12 ^bA^	27.44 ± 0.17 ^bB^
	24	25.94 ± 0.84 ^aC^	30.92 ± 0.25 ^aA^	29.64 ± 0.43 ^aAB^	28.67 ± 0.13 ^aB^
	72	25.78 ± 0.04 ^aB^	27.48 ± 0.58 ^bA^	26.42 ± 0.39 ^cB^	28.30 ± 0.15 ^aA^
Viscosity (unit: cp)	0	142.18 ± 1.91 ^aC^	162.73 ± 2.40 ^aB^	158.42 ± 0.67 ^aB^	683.48 ± 0.78 ^aA^
	24	74.45 ± 1.09 ^bD^	154.52 ± 1.08 ^bB^	110.59 ± 0.35 ^bC^	664.10 ± 2.36 ^bA^
	72	67.05 ± 1.68 ^cC^	113.28 ± 0.67 ^cB^	67.02 ± 1.62 ^cC^	494.69 ± 0.57 ^cA^

Note: abc represents the significant difference within groups (different fermentation time in the same group), and ABCD represented the significant difference between groups (same fermentation time in different groups). (*p* < 0.05). Values are expressed as averages ± standard deviation (n = 3).

**Table 3:** Changes of free amino acid contents in *P. yezoensis* sauces during fermentation (mg/g).

Groups	Taste	Fermentation Time/h	MMB-05	FJAT-7928	Mix	Control
Asp	sweet; umami	24	0.16 ± 0.00 ^cC^	0.24 ± 0.00 ^cB^	0.14 ± 0.00 ^cD^	0.50 ± 0.01 ^aA^
		48	0.18 ± 0.00 ^bD^	0.34 ± 0.00 ^bB^	0.18 ± 0.00 ^bC^	0.36 ± 0.00 ^bA^
		72	0.22 ± 0.00 ^aC^	0.52 ± 0.00 ^aA^	0.21 ± 0.00 ^aD^	0.22 ± 0.00 ^cB^
Thr	sweet	24	0.16 ± 0.00 ^cA^	0.05 ± 0.01 ^cC^	0.15 ± 0.00 ^cB^	0.05 ± 0.01 ^cC^
		48	0.18 ± 0.00 ^bA^	0.08 ± 0.00 ^bC^	0.15 ± 0.00 ^bB^	0.07 ± 0.00 ^bD^
		72	0.23 ± 0.00 ^aA^	0.12 ± 0.00 ^aC^	0.22 ± 0.00 ^aB^	0.09 ± 0.00 ^aD^
Ser	sweet	24	ND	0.06 ± 0.00 ^cA^	ND	0.06 ± 0.00 ^aA^
		48	ND	0.10 ± 0.00 ^bA^	ND	0.03 ± 0.00 ^bB^
		72	ND	0.12 ± 0.00 ^aA^	ND	0.03 ± 0.00 ^bB^
Glu	umami; sour	24	2.62 ± 0.00 ^cA^	2.22 ± 0.00 ^cC^	2.27 ± 0.00 ^cB^	2.21 ± 0.02 ^aC^
		48	2.76 ± 0.00 ^bB^	2.71 ± 0.02 ^bC^	3.08 ± 0.00 ^bA^	2.07 ± 0.00 ^bD^
		72	3.67 ± 0.01 ^aB^	3.86 ± 0.00 ^aA^	3.47 ± 0.00 ^aC^	1.76 ± 0.00 ^cD^
Gly	sweet	24	0.08 ± 0.00 ^cC^	0.08 ± 0.00 ^cD^	0.10 ± 0.00 ^bB^	0.10 ± 0.00 ^aA^
		48	0.11 ± 0.00 ^bA^	0.10 ± 0.00 ^bB^	0.10 ± 0.00 ^bB^	0.07 ± 0.00 ^cC^
		72	0.15 ± 0.00 ^aB^	0.15 ± 0.00 ^aB^	0.15 ± 0.00 ^aA^	0.07 ± 0.00 ^bC^
Ala	sweet	24	3.75 ± 0.00 ^cA^	2.68 ± 0.03 ^cC^	3.30 ± 0.00 ^cB^	2.68 ± 0.03 ^bC^
		48	3.90 ± 0.00 ^bB^	3.68 ± 0.03 ^bC^	3.97 ± 0.00 ^bA^	2.64 ± 0.00 ^bD^
		72	5.08 ± 0.01 ^aA^	4.34 ± 0.01 ^aC^	5.00 ± 0.00 ^aB^	2.85 ± 0.00 ^aD^
Cys		24	0.03 ± 0.00 ^cC^	0.04 ± 0.00 ^cB^	0.03 ± 0.00 ^cD^	0.09 ± 0.00 ^aA^
		48	0.04 ± 0.00 ^aC^	0.09 ± 0.00 ^bA^	0.03 ± 0.00 ^bD^	0.08 ± 0.00 ^aB^
		72	0.04 ± 0.00 ^bD^	0.19 ± 0.01 ^aA^	0.06 ± 0.00 ^aC^	0.08 ± 0.00 ^bB^
Val	sweet; bitter	24	0.01 ± 0.00 ^bC^	0.09 ± 0.00 ^cB^	0.01 ± 0.00 ^bC^	0.11 ± 0.00 ^aA^
		48	0.02 ± 0.00 ^aB^	0.11 ± 0.00 ^bA^	0.01 ± 0.00 ^bC^	0.11 ± 0.00 ^aA^
		72	0.02 ± 0.00 ^aC^	0.23 ± 0.00 ^aA^	0.02 ± 0.01 ^aC^	0.11 ± 0.00 ^aB^
Met	bitter	24	ND	ND	ND	ND
		48	0.01 ± 0.00 ^abB^	0.01 ± 0.00 ^bAB^	0.01 ± 0.00 ^aB^	0.01 ± 0.00 ^bA^
		72	0.01 ± 0.00 ^aC^	0.02 ± 0.00 ^aB^	0.01 ± 0.00 ^aC^	0.08 ± 0.00 ^aA^
Ile	bitter	24	0.02 ± 0.00 ^cB^	0.06 ± 0.00 ^cA^	0.02 ± 0.00 ^bB^	0.06 ± 0.00 ^aA^
		48	0.03 ± 0.00 ^bC^	0.08 ± 0.00 ^bA^	0.03 ± 0.00 ^aB^	0.03 ± 0.00 ^bB^
		72	0.03 ± 0.00 ^aB^	0.09 ± 0.00 ^aA^	0.03 ± 0.00 ^aB^	0.03 ± 0.00 ^cB^
Leu	bitter	24	0.02 ± 0.00 ^cD^	0.10 ± 0.00 ^bB^	0.04 ± 0.00 ^bC^	0.16 ± 0.00 ^cA^
		48	0.05 ± 0.00 ^bD^	0.16 ± 0.00 ^aB^	0.06 ± 0.00 ^aC^	0.16 ± 0.00 ^bA^
		72	0.06 ± 0.00 ^aB^	0.05 ± 0.00 ^cC^	0.06 ± 0.00 ^aB^	0.19 ± 0.00 ^aA^
Tyr	bitter	24	0.35 ± 0.00 ^cA^	0.36 ± 0.00 ^cA^	0.31 ± 0.01 ^cB^	0.03 ± 0.00 ^cC^
		48	0.37 ± 0.00 ^bC^	0.43 ± 0.00 ^bB^	0.45 ± 0.00 ^bA^	0.13 ± 0.00 ^bD^
		72	0.47 ± 0.00 ^aB^	0.49 ± 0.00 ^aA^	0.47 ± 0.00 ^aC^	0.27 ± 0.00 ^aD^
Phe	sweet; bitter	24	0.06 ± 0.00 ^cB^	0.04 ± 0.00^cC^	0.20 ± 0.00 ^cA^	0.04 ± 0.00 ^cC^
		48	0.07 ± 0.00 ^bC^	0.06 ± 0.00 ^bD^	0.27 ± 0.00 ^bA^	0.14 ± 0.00 ^bB^
		72	0.08 ± 0.00 ^aC^	0.07 ± 0.00 ^aD^	0.30 ± 0.00 ^aA^	0.30 ± 0.00 ^aB^
Lys	bitter	24	0.01 ± 0.00 ^cB^	0.01 ± 0.00 ^cB^	0.01 ± 0.00 ^cA^	0.01 ± 0.00 ^cB^
		48	0.02 ± 0.00 ^bA^	0.02 ± 0.00 ^bA^	0.02 ± 0.00 ^bB^	0.02 ± 0.00 ^bB^
		72	0.03 ± 0.00 ^aC^	0.08 ± 0.00 ^aA^	0.03 ± 0.00 ^aC^	0.05 ± 0.00 ^aB^
His	sour; bitter	24	1.37 ± 0.00 ^cB^	1.28 ± 0.00 ^cC^	1.42 ± 0.00 ^cA^	0.03 ± 0.00 ^bD^
		48	1.80 ± 0.00 ^aA^	1.65 ± 0.00 ^bC^	1.70 ± 0.00 ^bB^	0.03 ± 0.00 ^abD^
		72	1.75 ± 0.00 ^bC^	2.01 ± 0.00 ^aA^	2.00 ± 0.01 ^aB^	0.04 ± 0.00 ^aD^
Arg	bitter	24	0.17 ± 0.00 ^cA^	0.05 ± 0.00 ^cB^	ND	0.02 ± 0.00 ^cC^
		48	0.17 ± 0.00 ^bA^	0.07 ± 0.00 ^bB^	ND	0.02 ± 0.00 ^bC^
		72	0.22 ± 0.00 ^aA^	0.18 ± 0.00 ^aB^	0.01 ± 0.00 ^aD^	0.02 ± 0.00 ^aC^
Pro	sweet	24	0.28 ± 0.01 ^cA^	0.13 ± 0.00 ^cC^	0.23 ± 0.00 ^cB^	ND
		48	0.31 ± 0.00 ^bA^	0.18 ± 0.00 ^bC^	0.30 ± 0.01 ^bB^	ND
		72	0.36 ± 0.00 ^aA^	0.26 ± 0.00 ^aB^	0.34 ± 0.01 ^aA^	ND

Note: abc represents the significant difference within groups (different fermentation time in the same group), and ABCD represented the significant difference between groups (same fermentation time in different groups) (*p* < 0.05). Values are expressed as averages ± standard deviation (n = 3). ND: not detected.

**Table 4:** The relative content of volatile flavor compounds of *P. yezoensis* sauce after fermentation (72 h).

Compounds	CAS#	RI	Rt	Dt	Relative Amount (%)			
					MM B-05	FJAT-7928	Mix	Control
Aldehydes								
Phenylacetaldehyde	C122781	1763.10	2249.53	1.26	0.18 ± 0.03 ^A^	0.17 ± 0.01 ^A^	0.15 ± 0.01 ^A^	0.22 ± 0.02 ^A^
2-Decenal	C3913711	1766.10	2264.39	1.49	0.15 ± 0.01 ^A^	0.16 ± 0.01 ^A^	0.17 ± 0.03 ^A^	0.20 ± 0.01 ^A^
(E,E)-2,4-Octadienal	C30361285	1665.00	1818.42	1.28	0.21 ± 0.01 ^A^	0.22 ± 0.01 ^A^	0.21 ± 0.01 ^A^	0.29 ± 0.02 ^A^
Benzaldehyde	C100527	1549.30	1414.40	1.16	0.45 ± 0.01 ^C^	0.46 ± 0.03 ^C^	0.48 ± 0.02 ^B^	1.08 ± 0.02 ^A^
(E)-2-Octenal-M	C2548870	1436.10	1106.41	1.34	0.56 ± 0.01 ^AB^	0.54 ± 0.12 ^B^	0.73 ± 0.10 ^A^	0.90 ± 0.14 ^A^
(E)-2-Octenal-D	C2548870	1437.50	1109.70	1.82	0.07 ± 0.01 ^AB^	0.06 ± 0.01 ^B^	0.09 ± 0.02 ^A^	0.11 ± 0.01 ^A^
Nonanal	C124196	1400.90	1025.03	1.49	0.20 ± 0.01 ^B^	0.14 ± 0.01 ^C^	0.20 ± 0.01 ^B^	0.58 ± 0.03 ^A^
(E)-2-Heptenal-M	C18829555	1328.90	876.80	1.26	1.25 ± 0.03 ^C^	1.32 ± 0.14 ^C^	1.41 ± 0.04 ^B^	2.01 ± 0.10 ^A^
(E)-2-Heptenal-D	C18829555	1329.70	878.28	1.67	1.62 ± 0.06 ^B^	1.40 ± 0.45 ^B^	2.43 ± 0.44 ^A^	3.23 ± 0.55 ^A^
(E)-2-Hexenal-M	C6728263	1230.20	712.37	1.18	0.46 ± 0.02 ^C^	0.47 ± 0.04 ^C^	0.55 ± 0.02 ^B^	0.91 ± 0.03 ^A^
3-Methyl-2-butenal	C107868	1211.60	685.43	1.09	0.20 ± 0.00 ^C^	0.23 ± 0.00 ^B^	0.23 ± 0.00 ^A^	0.14 ± 0.01 ^D^
Cyclopentanone	C120923	1196.30	664.06	1.11	0.17 ± 0.00 ^B^	0.24 ± 0.01 ^A^	0.17 ± 0.00 ^B^	0.16 ± 0.00 ^C^
Heptanal-D	C111717	1194.30	661.28	1.69	0.02 ± 0.00 ^BC^	0.01 ± 0.00 ^C^	0.03 ± 0.00 ^B^	0.21 ± 0.02 ^A^
2-Heptanone-M	C110430	1191.70	657.56	1.26	0.35 ± 0.01 ^B^	0.49 ± 0.01 ^A^	0.35 ± 0.01 ^B^	0.36 ± 0.01 ^C^
2-Methyl-2-pentenal-M	C623369	1172.40	616.69	1.16	0.57 ± 0.00 ^A^	0.60 ± 0.00 ^A^	0.57 ± 0.02 ^A^	0.72 ± 0.00 ^A^
2-Methyl-2-pentenal-D	C623369	1172.90	617.61	1.50	0.93 ± 0.03 ^B^	0.81 ± 0.04 ^C^	0.80 ± 0.02 ^C^	1.69 ± 0.03 ^A^
(E)-2-Pentenal-M	C1576870	1148.30	569.31	1.11	0.76 ± 0.01 ^C^	0.81 ± 0.03 ^BC^	0.82 ± 0.01 ^A^	1.00 ± 0.01 ^AB^
(E)-2-Pentenal-D	C1576870	1148.30	569.31	1.36	1.30 ± 0.03 ^BC^	1.18 ± 0.16 ^C^	1.43 ± 0.09 ^AB^	1.90 ± 0.11 ^A^
(Z)-2-Pentenal-M	C1576869	1114.50	508.92	1.09	0.65 ± 0.00 ^C^	0.73 ± 0.03 ^B^	0.65 ± 0.03 ^BC^	1.19 ± 0.02 ^A^
(Z)-2-Pentenal-D	C1576869	1113.40	507.07	1.35	2.90 ± 0.01 ^A^	2.58 ± 0.05 ^C^	2.59 ± 0.02 ^B^	3.20 ± 0.01 ^B^
Hexanal-M	C66251	1098.70	482.91	1.28	0.46 ± 0.03 ^B^	0.19 ± 0.03 ^C^	0.55 ± 0.03 ^B^	1.04 ± 0.10 ^A^
Hexanal-D	C66251	1098.70	482.91	1.56	0.07 ± 0.01 ^BC^	0.02 ± 0.00 ^C^	0.11 ± 0.01 ^B^	0.39 ± 0.11 ^A^
Pentanal	C110623	1001.10	361.32	1.43	1.40 ± 0.10 ^C^	0.22 ± 0.06 ^D^	1.54 ± 0.08 ^B^	3.00 ± 0.06 ^A^
3-Methylbutanal	C590863	928.20	305.51	1.40	1.14 ± 0.05 ^B^	0.84 ± 0.10 ^C^	1.07 ± 0.03 ^B^	2.90 ± 0.02 ^A^
Butanal	C123728	891.90	281.52	1.28	0.39 ± 0.01 ^B^	0.33 ± 0.06 ^C^	0.44 ± 0.02 ^B^	1.03 ± 0.03 ^A^
Diethyl acetal	C105577	902.70	288.44	1.03	0.60 ± 0.02 ^B^	0.66 ± 0.03 ^B^	0.63 ± 0.03 ^B^	0.90 ± 0.01 ^A^
Acrolein	C107028	862.60	263.53	1.06	0.44 ± 0.01 ^B^	0.47 ± 0.01 ^B^	0.47 ± 0.02 ^A^	0.33 ± 0.02 ^C^
Propanal	C123386	812.10	235.20	1.14	2.97 ± 0.03 ^B^	3.23 ± 0.04 ^A^	2.85 ± 0.02 ^C^	3.76 ± 0.03 ^B^
Acetaldehyde	C75070	756.90	207.69	1.02	0.82 ± 0.05 ^A^	0.58 ± 0.04 ^B^	0.79 ± 0.09 ^A^	0.57 ± 0.03 ^B^
2-Methylpropanal	C78842	828.40	243.99	1.28	0.01 ± 0.00 ^BC^	0.01 ± 0.00 ^C^	0.02 ± 0.00 ^B^	0.08 ± 0.00 ^A^
Octanal-M	C124130	1294.20	813.28	1.41	0.09 ± 0.00 ^B^	0.10 ± 0.01 ^B^	0.10 ± 0.00 ^B^	0.47 ± 0.26 ^A^
Octanal-D	C124130	1294.30	813.41	1.83	0.02 ± 0.00 ^B^	0.02 ± 0.00 ^B^	0.02 ± 0.00 ^B^	0.07 ± 0.04 ^A^
(E)-2-Hexenal-D	C6728263	1230.10	712.13	1.52	0.32 ± 0.00 ^A^	0.25 ± 0.05 ^B^	0.33 ± 0.02 ^A^	0.44 ± 0.04 ^A^
Methacrolein	C78853	891.30	281.10	1.22	0.26 ± 0.01 ^B^	0.24 ± 0.03 ^BC^	0.33 ± 0.01 ^A^	0.26 ± 0.02 ^C^
Ketones								
2-Cyclohexen-1-one	C930687	1423.60	1076.91	1.10	0.03 ± 0.00 ^A^	0.03 ± 0.00 ^A^	0.03 ± 0.00 ^A^	0.02 ± 0.00 ^B^
6-Methyl-5-hepten-2-one	C110930	1345.00	907.92	1.18	0.21 ± 0.01 ^B^	0.17 ± 0.01 ^C^	0.24 ± 0.02 ^A^	0.16 ± 0.01 ^D^
1-Hydroxy-2-propanone-M	C116096	1314.10	849.09	1.07	1.87 ± 0.02 ^A^	1.93 ± 0.04 ^A^	1.90 ± 0.03 ^A^	1.92 ± 0.25 ^B^
1-Hydroxy-2-propanone-D	C116096	1314.80	850.42	1.23	0.94 ± 0.07 ^A^	0.98 ± 0.02 ^A^	0.93 ± 0.02 ^A^	1.04 ± 0.07 ^B^
3-Hydroxy-2-butanone	C513860	1298.70	821.10	1.33	6.05 ± 0.17 ^A^	5.84 ± 0.09^A^	6.00 ± 0.10 ^A^	1.68 ± 1.74 ^B^
1-Octen-3-one-M	C4312996	1312.70	846.42	1.28	0.88 ± 0.03 ^A^	0.79 ± 0.01 ^B^	0.87 ± 0.02 ^A^	0.85 ± 0.11 ^B^
1-Octen-3-one-D	C4312996	1308.30	838.43	1.68	0.29 ± 0.01 ^AB^	0.24 ± 0.11 ^B^	0.43 ± 0.11 ^A^	0.56 ± 0.17 ^A^
Cyclohexanone	C108941	1311.90	845.09	1.46	0.27 ± 0.03 ^B^	0.3 ± 0.06 ^B^	0.34 ± 0.03 ^AB^	0.49 ± 0.04 ^A^
Heptanal-M	C111717	1195.00	662.20	1.34	0.21 ± 0.01 ^C^	0.13 ± 0.01 ^D^	0.23 ± 0.01 ^B^	0.60 ± 0.02 ^A^
2-Heptanone-D	C110430	1191.30	656.63	1.63	0.18 ± 0.01 ^B^	0.28 ± 0.03 ^A^	0.16 ± 0.01 ^B^	0.22 ± 0.01 ^B^
2,3-Pentanedione	C600146	1061.30	431.90	1.20	0.62 ± 0.05 ^BC^	0.53 ± 0.10 ^C^	0.72 ± 0.07 ^AB^	1.03 ± 0.10 ^A^
1-Penten-3-one-M	C1629589	1038.90	404.22	1.08	0.46 ± 0.01 ^B^	0.51 ± 0.00 ^A^	0.46 ± 0.01 ^B^	0.63 ± 0.00 ^A^
1-Penten-3-one-D	C1629589	1038.50	403.76	1.31	1.49 ± 0.04 ^AB^	1.42 ± 0.20 ^B^	1.66 ± 0.10 ^A^	1.93 ± 0.10 ^AB^
2-Pentanone	C107879	997.20	357.17	1.36	1.44 ± 0.01 ^B^	1.80 ± 0.10 ^A^	1.49 ± 0.03 ^B^	1.51 ± 0.02 ^C^
2-Butanone	C78933	914.60	296.28	1.24	3.48 ± 0.04 ^A^	3.67 ± 0.07 ^A^	3.47 ± 0.01 ^A^	4.32 ± 0.04 ^A^
Acetone	C67641	835.90	248.15	1.11	5.42 ± 0.18 ^AB^	6.09 ± 0.38 ^A^	5.27 ± 0.07 ^B^	7.13 ± 0.04 ^AB^
Alcohols								
1-Octanol	C111875	1652.70	1770.52	1.48	0.18 ± 0.04 ^A^	0.18 ± 0.01 ^A^	0.18 ± 0.02 ^A^	0.19 ± 0.00 ^A^
Linalool	C78706	1633.00	1696.19	1.22	0.15 ± 0.02 ^A^	0.17 ± 0.01 ^A^	0.15 ± 0.01 ^A^	0.14 ± 0.01 ^B^
1-Octen-3-ol	C3391864	1483.40	1225.97	1.17	0.14 ± 0.01 ^BC^	0.13 ± 0.02 ^C^	0.18 ± 0.03 ^AB^	0.26 ± 0.04 ^A^
1-Heptanol	C111706	1486.60	1234.50	1.41	0.07 ± 0.00 ^B^	0.08 ± 0.00 ^A^	0.08 ± 0.00 ^AB^	0.06 ± 0.00 ^C^
(Z)-Hex-3-enol	C928961	1418.70	1065.36	1.25	0.11 ± 0.01 ^A^	0.09 ± 0.01 ^B^	0.12 ± 0.01 ^A^	0.05 ± 0.01 ^C^
1-Hexanol-M	C111273	1369.20	956.84	1.33	1.21 ± 0.01 ^B^	1.39 ± 0.05 ^A^	1.12 ± 0.02 ^C^	0.19 ± 0.03 ^D^
1-Hexanol-D	C111273	1368.40	955.36	1.64	0.22 ± 0.00 ^B^	0.28 ± 0.02 ^A^	0.20 ± 0.00 ^C^	0.04 ± 0.01 ^D^
(Z)-2-Penten-1-ol	C1576950	1337.40	893.10	0.95	0.59 ± 0.00 ^A^	0.52 ± 0.02 ^B^	0.57 ± 0.01 ^A^	0.50 ± 0.00 ^C^
1-Pentanol-M	C71410	1264.70	765.11	1.26	0.98 ± 0.02 ^B^	1.24 ± 0.04^A^	0.98 ± 0.02 ^B^	1.51 ± 0.04 ^A^
1-Pentanol-D	C71410	1263.90	763.78	1.51	0.37 ± 0.02 ^B^	0.62 ± 0.06 ^A^	0.39 ± 0.03 ^B^	0.77 ± 0.06 ^A^
3-Methyl-3-buten-1-ol-M	C763326	1261.30	759.75	1.17	0.35 ± 0.01 ^A^	0.23 ± 0.03 ^B^	0.33 ± 0.01 ^A^	0.07 ± 0.00 ^C^
3-Methyl-3-buten-1-ol-D	C763326	1261.30	759.75	1.44	0.34 ± 0.01 ^A^	0.27 ± 0.01 ^B^	0.35 ± 0.01 ^A^	0.12 ± 0.00 ^C^
3-Methyl-1-butanol-M	C123513	1220.10	697.51	1.24	1.19 ± 0.01 ^A^	1.29 ± 0.02 ^A^	1.21 ± 0.00 ^A^	0.40 ± 0.07 ^B^
3-Methyl-1-butanol-D	C123513	1219.40	696.58	1.49	3.43 ± 0.03 ^A^	3.04 ± 0.01 ^B^	2.71 ± 0.01 ^C^	0.15 ± 0.02 ^D^
1-Penten-3-ol-M	C616251	1176.50	625.05	0.95	0.82 ± 0.01 ^B^	0.86 ± 0.00 ^B^	0.86 ± 0.02 ^A^	0.98 ± 0.01 ^C^
1-Penten-3-ol-D	C616251	1175.10	622.26	1.37	0.58 ± 0.01 ^B^	0.55 ± 0.01 ^C^	0.53 ± 0.00 ^C^	0.80 ± 0.03 ^A^
1-Butanol-M	C71363	1160.80	593.46	1.18	1.02 ± 0.01 ^B^	1.12 ± 0.03 ^A^	1.01 ± 0.01 ^B^	0.81 ± 0.01 ^C^
1-Butanol-D	C71363	1160.80	593.46	1.38	0.75 ± 0.01 ^B^	0.87 ± 0.01 ^A^	0.75 ± 0.01 ^B^	0.27 ± 0.01 ^C^
2-Methyl-1-propanol-M	C78831	1105.60	494.06	1.17	0.55 ± 0.01 ^B^	0.64 ± 0.01 ^A^	0.60 ± 0.01 ^A^	0.27 ± 0.01 ^C^
2-Methyl-1-propanol-D	C78831	1107.90	497.78	1.37	1.00 ± 0.02 ^A^	0.93 ± 0.03 ^B^	0.79 ± 0.02 ^C^	0.13 ± 0.02 ^D^
1-Propanol-M	C71238	1051.40	419.45	1.11	0.49 ± 0.01 ^B^	0.80 ± 0.04^A^	0.45 ± 0.01 ^B^	0.38 ± 0.02 ^C^
1-Propanol-D	C71238	1052.90	421.29	1.25	0.27 ± 0.00 ^B^	0.76 ± 0.02 ^A^	0.26 ± 0.01 ^B^	0.15 ± 0.01 ^C^
2-Butanol	C78922	1034.70	399.15	1.15	0.15 ± 0.00 ^A^	0.15 ± 0.00 ^B^	0.14 ± 0.00 ^C^	0.10 ± 0.00 ^D^
Ethanol	C64175	941.40	314.73	1.13	4.62 ± 0.04 ^AB^	4.83 ± 0.07 ^A^	4.49 ± 0.04 ^B^	3.42 ± 0.03 ^C^
Esters								
γ-Butyrolactone	C96480	1709.70	2003.42	1.09	0.75 ± 0.03 ^A^	0.69 ± 0.02 ^B^	0.65 ± 0.06 ^B^	0.78 ± 0.03 ^B^
Ethyl pyruvate	C617356	1249.40	741.17	1.15	0.24 ± 0.00 ^B^	0.23 ± 0.02 ^B^	0.27 ± 0.01 ^B^	1.68 ± 0.07 ^A^
Ethyl hexanoate	C123660	1244.50	733.73	1.34	0.17 ± 0.01 ^B^	0.19 ± 0.01 ^B^	0.18 ± 0.01 ^AB^	0.25 ± 0.00 ^A^
Ethyl butanoate-M	C105544	1049.50	417.14	1.20	0.42 ± 0.01 ^A^	0.32 ± 0.01 ^C^	0.37 ± 0.00 ^B^	0.44 ± 0.02 ^B^
Ethyl butanoate-D	C105544	1049.20	416.68	1.56	0.09 ± 0.01 ^B^	0.08 ± 0.02 ^BC^	0.07 ± 0.01 ^C^	0.17 ± 0.01 ^A^
Ethyl Acetate	C141786	894.10	282.91	1.34	0.47 ± 0.01 ^A^	0.39 ± 0.01 ^B^	0.38 ± 0.01 ^B^	0.38 ± 0.01 ^C^
Methyl acetate	C79209	878.50	273.15	1.19	0.34 ± 0.01 ^A^	0.31 ± 0.04 ^B^	0.34 ± 0.02 ^A^	0.17 ± 0.01 ^C^
Ethyl isobutyrate	C97621	963.80	331.00	1.19	0.36 ± 0.01 ^B^	0.3 ± 0.03 ^C^	0.37 ± 0.01 ^B^	0.65 ± 0.02 ^A^
Acids								
2-Methylpropanoic acid	C79312	1690.30	1920.83	1.17	0.15 ± 0.01 ^A^	0.16 ± 0.01 ^A^	0.15 ± 0.00 ^A^	0.09 ± 0.02 ^B^
Propanoic acid	C79094	1637.00	1711.06	1.12	1.20 ± 0.11 ^B^	1.62 ± 0.04 ^A^	1.31 ± 0.02 ^B^	0.5 ± 0.11^C^
Acetic acid-M	C64197	1500.50	1272.51	1.06	9.55 ± 0.14 ^A^	9.76 ± 0.15 ^A^	9.13 ± 0.15 ^A^	7.93 ± 1.23 ^B^
Acetic acid-D	C64197	1502.20	1277.05	1.15	10.63 ± 0.26 ^A^	10.54 ± 0.03 ^A^	11.29 ± 0.59 ^A^	4.53 ± 2.05 ^B^
Furans								
Dihydro-5-methyl-2(3H)-furanone	C108292	1735.90	2120.69	1.13	0.18 ± 0.02 ^A^	0.17 ± 0.02 ^AB^	0.16 ± 0.01 ^AB^	0.19 ± 0.01 ^B^
2-Acetylfuran	C1192627	1539.20	1383.76	1.13	0.33 ± 0.00 ^A^	0.26 ± 0.01 ^C^	0.25 ± 0.00 ^C^	0.36 ± 0.01 ^B^
5-Methyl-2(3H)-furanone	C591128	1442.90	1122.86	1.13	0.05 ± 0.00 ^A^	0.05 ± 0.00 ^B^	0.05 ± 0.00 ^A^	0.04 ± 0.00 ^C^
2-Pentylfuran	C3777693	1239.00	725.37	1.25	0.23 ± 0.02 ^A^	0.21 ± 0.02 ^A^	0.16 ± 0.02 ^B^	0.07 ± 0.01 ^C^
2-Ethylfuran	C3208160	968.60	334.57	1.04	0.89 ± 0.04 ^B^	1.02 ± 0.04 ^A^	0.82 ± 0.03 ^B^	0.77 ± 0.03 ^C^
Furfural	C98011	1491.90	1248.75	1.09	0.74 ± 0.02 ^A^	0.74 ± 0.01 ^A^	0.66 ± 0.01 ^B^	0.91 ± 0.04 ^A^
Pyrazines								
2,6-Dimethyl-3-ethylpyrazine	C13925070	1557.00	1438.52	1.24	0.14 ± 0.01 ^A^	0.17 ± 0.01 ^A^	0.14 ± 0.00 ^A^	0.11 ± 0.02 ^B^
2-Ethyl-5-methylpyrazine	C13360640	1393.50	1008.72	1.15	0.05 ± 0.00 ^A^	0.04 ± 0.00 ^B^	0.05 ± 0.00 ^A^	0.03 ± 0.00 ^C^
Ethylpyrazine	C13925003	1369.20	956.84	1.11	0.12 ± 0.00 ^B^	0.11 ± 0.00 ^C^	0.12 ± 0.00 ^BC^	0.18 ± 0.02 ^A^
2,5-Dimethylpyrazine	C123320	1350.20	918.30	1.12	0.26 ± 0.01 ^B^	0.28 ± 0.01 ^B^	0.26 ± 0.01 ^B^	0.37 ± 0.02 ^A^
2-Methylpyrazine	C109080	1274.70	781.11	1.08	0.58 ± 0.01 ^A^	0.53 ± 0.01 ^B^	0.51 ± 0.01 ^B^	0.59 ± 0.01 ^C^
Aromatic hydrocarbons								
p-Xylene	C106423	1143.90	560.95	1.08	0.12 ± 0.01 ^B^	0.11 ± 0.00 ^C^	0.12 ± 0.00 ^BC^	0.18 ± 0.00 ^A^
Toluene	C108883	1062.30	433.19	1.04	0.60 ± 0.01 ^B^	0.60 ± 0.01 ^C^	0.60 ± 0.00 ^B^	0.77 ± 0.01 ^A^
Pyridines								
3-Ethylpyridine	C536787	1384.60	989.45	1.10	0.06 ± 0.00 ^A^	0.06 ± 0.00 ^A^	0.06 ± 0.00 ^A^	0.05 ± 0.00 ^B^
Others								
α-Terpinene	C99865	1189.60	652.91	1.23	0.18 ± 0.01 ^A^	0.16 ± 0.00 ^B^	0.14 ± 0.00 ^C^	0.19 ± 0.00 ^B^
α-Phellandrene	C99832	1175.10	622.26	1.67	0.14 ± 0.00 ^B^	0.13 ± 0.00 ^C^	0.13 ± 0.01 ^C^	0.21 ± 0.01 ^A^
β-Pinene	C127913	1143.90	560.95	1.22	0.29 ± 0.00 ^A^	0.25 ± 0.00 ^C^	0.23 ± 0.01 ^C^	0.32 ± 0.00 ^B^
Tetrahydrothiophene-M	C110010	1121.10	520.07	1.05	0.58 ± 0.01 ^B^	0.61 ± 0.01 ^B^	0.59 ± 0.01 ^B^	0.78 ± 0.01 ^A^
Tetrahydrothiophene-D	C110010	1121.10	520.07	1.31	0.35 ± 0.00 ^A^	0.31 ± 0.01 ^B^	0.31 ± 0.01 ^B^	0.44 ± 0.03 ^A^
Dimethyl disulfide	C624920	1081.60	458.76	1.14	0.11 ± 0.01 ^BC^	0.09 ± 0.02 ^C^	0.15 ± 0.03 ^B^	0.34 ± 0.04 ^A^
Thiophene	C110021	1028.00	391.31	1.04	1.58 ± 0.01 ^A^	1.65 ± 0.03 ^A^	1.45 ± 0.06 ^B^	1.70 ± 0.05 ^C^
Acrylonitrile	C107131	1011.70	372.86	1.08	0.39 ± 0.00 ^B^	0.43 ± 0.02 ^A^	0.39 ± 0.00 ^B^	0.42 ± 0.00 ^C^
Dimethyl sulfide	C75183	788.30	222.92	1.09	2.24 ± 0.02 ^A^	1.92 ± 0.11 ^B^	2.21 ± 0.00 ^A^	2.22 ± 0.05 ^B^

Note: Compounds percentage of total area of identified volatile compounds. RI is the retention index, Rt is retention time, Dt is migration time. ABCD represented the significant difference between groups (same fermentation time in different groups) (*p* < 0.05). Values are expressed as averages ± standard deviation (n = 3).

## Data Availability

The data presented in this study are available on request from the corresponding author.

## Supplementary Materials

The following supporting information can be downloaded at: https://www.mdpi.com/article/10.3390/foods11182875/s1, Figure S1: Phylogenetic tree of *L. plantarum* MMB-05 based on 16S rRNA gene sequences; Figure S2: Colony morphology and Gram staining of *L. plantarum* MMB-05; Table S1: Changes of viable cell counts in *P. yezoensis* sauce fermentation with different LAB strains.
