# Supplementary material for: Screening of a Novel Lactiplantibacillus plantarum MMB-05 and Lacticaseibacillus casei Fermented Sandwich Seaweed Scraps: Chemical Composition, In Vitro Antioxidant, and Volatile Compounds Analysis by GC-IMS
Source: Foods. 2022 Sep 16;11(18):2875. doi: 10.3390/foods11182875 (PMC9498330; doi:10.3390/foods11182875)
Supplement: Supplementary file 1 [file foods-11-02875-s001.zip › foods-1887570-supplementary.pdf]

Screening of a Novel *Lactiplantibacillus plantarum* MMB-05 and *Lacticaseibacillus casei* Fermented Sandwich Seaweed Scraps: Chemical Composition, *In Vitro* Antioxidant, and Volatile Compounds Analysis by GC-IMS

Tengqi Gao [1, 2], Jinling Chen[1], Jie Xu [3], Han Gu[1], Pengpeng Zhao[1], Wenbin Wang[1, 2], Saikun Pan[1, 2], Yang Tao[4], Hongli Wang [1], Jie Yang[1, 2*]

[1]Jiangsu Key Laboratory of Marine Bioresources and Environment/Jiangsu Key Laboratory of Marine Biotechnology, Jiangsu Ocean University, Lianyungang, 222005, China

[2]Co-Innovation Center of Jiangsu Marine Bio-industry Technology, Jiangsu Ocean University, Lianyungang 222005, China

[3]Anqiu Agricultural Product Quality and Safety Management Service Center, AnQiu, 262100, China

[4]College of Food Science and Technology, Nanjing Agricultural University, 1 Weigang, Nanjing 210095, China

*Corresponding authors:

1. Hongli Wang, Email: 18221338903@163.com

Jiangsu Key Laboratory of Marine Bioresources and Environment/Jiangsu Key Laboratory of Marine Biotechnology, Jiangsu Ocean University, Lianyungang, 222005, China

2. Jie Yang, Email: yangjie0737@163.com

Jiangsu Key Laboratory of Marine Bioresources and Environment/Jiangsu Key Laboratory of Marine Biotechnology, Jiangsu Ocean University, Lianyungang, 222005, China

Table S1. Changes of viable cell counts in *P. yezoensis* sauce fermentation with different LAB strains.

Numbers	viable cell counts (Log CFU/mL)			
	0 h	24 h	72 h	96 h
L1	6.75 ± 0.03	8.45 ± 0.03	9.86 ± 0.04	8.37 ± 0.04
L2	6.22 ± 0.13	8.66 ± 0.07	9.71 ± 0.12	8.48 ± 0.06
L3	6.35 ± 0.01	9.03 ± 0.11	9.98 ± 0.05	8.36 ± 0.07
L4	6.98 ± 0.02	9.34 ± 0.09	10.65 ± 0.06	9.14 ± 0.09
MMB-05	7.01 ± 0.18	13.11 ± 0.06	11.92 ± 0.11	11.43 ± 0.08
L6	7.61 ± 0.12	10.16 ± 0.04	11.24 ± 0.03	10.13 ± 0.03
L7	7.33 ± 0.02	9.56 ± 0.01	10.97 ± 0.04	9.57 ± 0.08
L8	6.78 ± 0.09	8.85 ± 0.05	9.82 ± 0.02	8.32 ± 0.05
L9	6.45 ± 0.12	8.63 ± 0.05	9.77 ± 0.02	8.67 ± 0.11
L10	7.15 ± 0.03	9.01 ± 0.02	10.01 ± 0.01	9.13 ± 0.04

Values are expressed as averages ± standard deviation (n = 3).

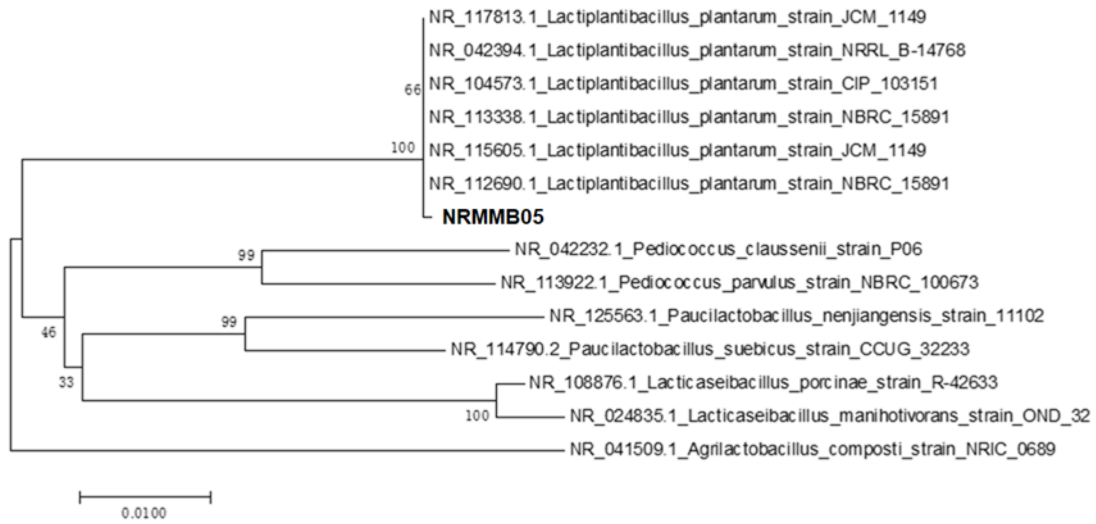

Figure S1. Phylogenetic tree of *L. plantarum* MMB-05 based on 16S rRNA gene sequences.

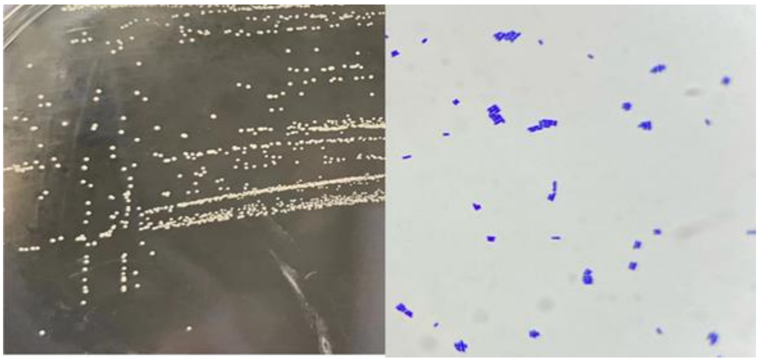

Figure S2. Colony morphology and Gram staining of *L. plantarum* MMB-05.
